# Hawaiian Plants with Beneficial Effects on Sleep, Anxiety, and Mood, etc.

**DOI:** 10.3390/ph16091228

**Published:** 2023-08-30

**Authors:** Pornphimon Meesakul, Tyler Shea, Shi Xuan Wong, Yutaka Kuroki, Shugeng Cao

**Affiliations:** 1 Department of Pharmaceutical Sciences, Daniel K. Inouye College of Pharmacy, University of Hawai’i at Hilo, 200 W. Kawili St., Hilo, HI 96720, USA; pmeesak@hawaii.edu; 2 Chemistry Department, University of Hawai’i at Hilo, 200 W. Kawili St., Hilo, HI 96720, USA; tylerms3@hawaii.edu; 3 Delightex Pte. Ltd., 230 Victoria Street, #15-01/08 Bugis Junction Towers, Singapore 188024, Singapore; shixuan@delightexplorers.com (S.X.W.); yutaka@delightexplorers.com (Y.K.)

**Keywords:** Hawaiian plants, sleep, anxiety, mood, pain

## Abstract

Diverse chemical messengers are responsible for maintaining homeostasis in the human body, for example, hormones and neurotransmitters. Various Hawaiian plant species produce compounds that exert effects on these messengers and the systems of which they are a part. The main purpose of this review article is to evaluate the potential effects of Hawaiian plants on reducing pain and anxiety and improving sleep and mood. A comprehensive literature search was conducted in SciFinder, PubMed, Science Direct, Scopus, Google Scholar, and Scientific Information Database between 2019 and 2023 to identify related articles. Results indicate that several Hawaiian plant species, such as *M. citrifolia* and *P. methysticum*, have medicinal properties associated with these effects. These plants have been used in traditional Hawaiian cultural practices for centuries, suggesting their potential to benefit human health and well-being. This review presents a comprehensive analysis of the available evidence concerning the potential impacts of Hawaiian plants on sleep, anxiety, mood, and pain.

## 1. Introduction

Hormones are chemical messengers in the endocrine system. They are secreted by endocrine organs and subsequently circulate uninhibitedly throughout the bloodstream. Some hormones also have autocrine and paracrine effects. Hormones play diverse roles in the body, such as regulating sleep (melatonin), blood sugar (insulin), or sexual maturation (estrogen/testosterone). Plant metabolites that affect hormones can then affect the body systems regulated by those hormones. In contrast, neurotransmitters are secreted into the synaptic cleft of nerve junctions, regulating the action potential of the proximal neuron. As with hormones, plant metabolites that affect neurotransmitters can have powerful physiological effects. Additionally, the nervous and endocrine systems interact in complex ways, offering many opportunities for beneficial intervention. Some hormones and neurotransmitters include dopamine, serotonin, endorphins, oxytocin, gamma-aminobutyric acid (GABA), melatonin, dopamine, etc., each of which has the ability to create positive emotions [1,2,3,4]. Serotonin, for example, is responsible for a variety of functions, including elevating mood and reducing symptoms of depression [5].

Plants may have a variety of beneficial impacts on pain, sleep, anxiety, and mood. For instance, the scents of certain plants, such as lavender, chamomile, rose, and orange oil, have been known to have calming effects [5,6,7,8,9,10]. Additionally, some plants are capable of producing essential oils that have demonstrated the capacity to exert beneficial effects on an individual’s mental well-being [6,8,9,10]. Furthermore, some plants contain secondary metabolites that can interact with the human body in specific ways. For example, the opium poppy contains diverse alkaloids, some of which can reduce pain and anxiety and induce relaxation and sedation, as well as feelings of euphoria [11].

Endemic and introduced plants have a long history of medicinal use amongst the various tribes in Hawaii. These plants provide a range of benefits, as well as being an important part of the traditional diet prior to Western contact [12,13]. With the breakdown of cultural traditions and the introduction of Western medicine, herbal treatment is much less common in modern society. However, in recent times, indigenous plants have once again come to the forefront as a potential solution to improve health for current generations.

Many plant species have been traditionally used for medicinal purposes. The exact number of plants used in Hawaiian traditional medicine can vary depending on various sources and cultural practices. However, it is estimated that hundreds of plant species have been traditionally employed for their medicinal properties. The percentage of medicinal plant use in the region is difficult to quantify precisely. Traditional medicine practices in Hawaii, like in many other cultures, have evolved over time and have been influenced by numerous factors such as Westernization and modern healthcare practices.

The Hawaiian naturalized vascular plants checklist [14] has identified 1652 species in Hawaii, while the Hawaiian medicinal plants list [12] has recorded 1729 native species. Interestingly, only 166 of these species (9.6%) have been recognized as Hawaiian medicinal plants. Our literature search led to the identification of fourteen plant species including *Acacia koa*, *Curcuma longa*, *Dodonaea viscosa*, *Mimosa pudica*, *Piper methysticum*, *Stachytarpheta cayennensis*, *Azadirachta indica*, *Cocos nucifera*, *Morinda citrifolia*, *Passiflora edulis*, *Pipturus albidus*, *Rhodiola rosea*, *Aloe vera*, and *Argyreia speciosa*, which were discovered in Hawaii that have been reported to affect sleep, mood, anxiety, and pain. According to the University of Hawaii database of Native Plants Hawaii, the plants listed below are native to Hawaii (*A. koa*, *D. viscosa*, and *P. albidus*). The other remaining plants were introduced to Hawaii, and they grow very well in the Aloha State. Some of these species have yet to be studied for their potential benefits in addressing sleep, mood disturbances, anxiety, and pain. 

**Figure 1:**
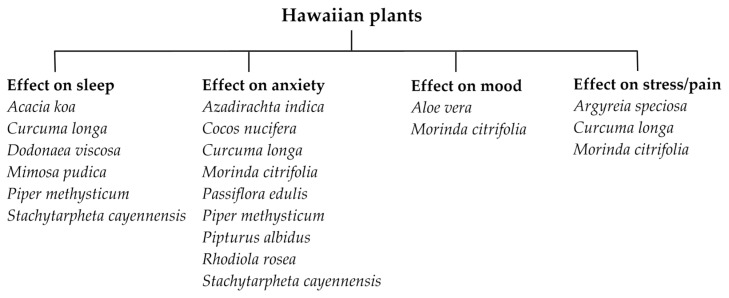
A list of Hawaiian plants with effects on sleep, anxiety, mood, and stress/pain.

This review article summarizes the potential benefits of utilizing Hawaiian plants for treating sleep and mood disturbances, anxiety, and pain (Figure 1). We also discuss the importance of Hawaiian plants in traditional Hawaiian healing practices, as well as the potential side effects associated with their use. Our review suggests that Hawaiian plants may have beneficial effects on a wide range of conditions.

## 2. Results and Discussions

### 2.1. Hawaiian Plants with Beneficial Effects on Sleep (Table 1)

#### 2.1.1. *Acacia koa* A. Gray (Hawaiian Name: Koa)

*Acacia koa* Gray or koa, a large tree belonging to the Fabaceae family, is most distributed on Hawaii islands [15]. In 2001, Krauss reported that in the context of traditional usage, *A. koa* displays noteworthy sleep-inducing attributes when administered to individuals who are contending with fever and enduring extended periods of confinement to a bed [16]. However, based on the existing corpus of scientific literature, no studies or reports have been documented to establish any sleep-related effects linked to the *A. koa* tree.

#### 2.1.2. *Curcuma longa* L. (Turmeric, Indian Saffron, Hawaiian Name: Ōlena)

**Figure 2:**
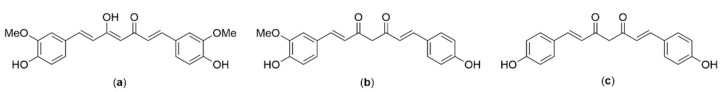
Major compounds from *C. longa*: (**a**) curcumin, (**b**) demethoxycurcumin, and (**c**) bisdemethoxycurcumin.

*Curcuma longa* (turmeric) is a flowering plant belonging to the ginger family, Zingiberaceae, the rhizomes of which are widely used in cooking in many countries. In 2020, Erfanizadeh et al. evaluated the effect of chronic sleep deprivation (CSD) in rat models on the superior cervical ganglion (SCG) histomorphometrical changes and the protective effect of curcumin (Figure 2) from *C. longa* in preventing these changes. Male rats (totaling thirty-six) were assigned randomly to six different groups: control, curcumin, Chronic sleep deprivation (CSD), chronic sleep deprivation with curcumin supplementation (CSD + curcumin), control with a grid floor, and grid floor with curcumin supplementation. The induction of CSD involved using a modified multiple-platform setup for 21 consecutive days. At the end of the sleep deprivation period or the assigned treatment duration, the rats were sacrificed. During the sacrifice, the SCG was extracted from each rat for subsequent stereological and TUNEL evaluations, as well as for assessing the spatial arrangement of neurons within the SCG structure. This study found that chronic sleep deprivation (CSD) led to a significant decrease in the volume of the superior cervical ganglion (SCG), as well as in the number of neurons and satellite glial cells it contained compared to the control group (*p* < 0.05). Administering curcumin during CSD prevented these reductions. Additionally, CSD caused notable apoptosis in SCG cells; however, curcumin treatment significantly reduced this apoptosis (*p* < 0.01), maintaining a regular cell pattern. CSD disrupted the spatial arrangement of ganglionic neurons, while curcumin preserved their normal organization. CSD might induce the loss of neurons and alterations in the structure, including a haphazard spatial arrangement within the neurons of the superior cervical ganglion. The harmful consequences of sleep deprivation may be averted by taking curcumin orally. Additionally, the ingestion of curcumin by an individual in good health could potentially result in diminished cellular demise [17]. 

Ishola et al. investigated the effects of administering *C. longa* rhizome to mice at varying doses (100, 200 or 400 mg/kg, p.o.) before subjecting them to behavioral tests, including elevated plus maze (EPM), hole board test (HBT), open field test (OFT), and rotarod test assessing anxiolytic and hypnotic responses. Additionally, the impact of *C. longa* rhizome on hexobarbitone-induced sleeping time (HIST) was examined as a measure of hypnotic activity. The study also investigated the role of the GABAergic and nitrergic systems in mediating the anxiolytic and hypnotic effects of *C. longa*. Additionally, interactions of *C. longa* with midazolam, imipramine, nifedipine, propranolol, and carbamazepine were studied in these models. The results indicated that the most pronounced anxiolytic-like impact was achieved with a 400 mg/kg dose in the EPM and hole-board tests, while muscle coordination remained unaffected in the rotarod test. Notably, the most significant enhancement of hypnosis was observed at a 100 mg/kg dose. These effects were found to be linked to interactions with the GABAergic and nitrergic systems. Moreover, the study revealed that co-administration of Curcuma longa with midazolam heightened barbiturate-induced hypnosis. In essence, *C. longa* exhibited anxiolytic and hypnotic properties, shedding light on its potential therapeutic applications. This study found that *C. longa* rhizome-induced anxiolytic-hypnotic-like effects were reversed by the pretreatment of mice with flumazenil or N^G^-nitro-l-arginine, which means *C. longa* possesses anxiolytic and hypnotic effects through its interaction with GABAergic and nitrergic systems [18]. 

In 2021, Um et al. conducted a study to assess the hypnotic effects of *C. longa* roots extract compared to diazepam (positive control) on sleep in mice, using the pentobarbital-induced sleep test and electroencephalography (EEG) recordings to analyze sleep-wake patterns. The research also aimed to investigate the molecular mechanism underlying *C. longa* roots extract’s sleep-inducing effects using ex vivo electrophysiological recordings from brain slices in mice lacking the histamine H_1_ receptor (H_1_R knockout mice). The results showed that both oral administration of *C. longa* roots extract (at doses of 10–100 mg/kg) and diazepam (2 or 6 mg/kg) led to a significant reduction in sleep latency and an increase in non-rapid eye movement sleep (NREMS) duration in mice. Notably, this increase in NREMS duration was observed without delta activity. Similar to doxepin and *C. longa* roots extract, it exhibited an inhibitory effect on the increase in action potentials induced by the histamine H_1_ receptor agonist (2-pyridylethylamine dihydrochloride) in hypothalamic neurons. Furthermore, the effects of *C. longa* root extract in animal tests with neurotransmitter agonists or antagonists resembled the H_1_ receptor antagonistic effects of doxepin. Both *C. longa* root extract and doxepin demonstrated a reduction in sleep latency and an increase in NREMS in wild-type mice; however, these effects were not observed in mice lacking the H_1_ receptor [19].

In 2022, Um et al. mentioned that the sedative-hypnotic effect of *C. longa* extract has been reported, and although the sleep-promoting compounds within *C. longa* extract have not yet been demonstrated, curcuminoids (curcumin, demethoxycurcumin, and bisdemethoxycurcumin) (Figure 2), which are major constituents of *C. longa* extract responsible for various biological activities, are initially hypothesized to be the sedative-hypnotic compounds of *C. longa* extract. In this study, the effects and underlying mechanisms of curcuminoids and their individual constituents on the sleep-wake cycle of mice were investigated. Molecular docking studies, histamine H_1_ receptor (H_1_R) binding assays, and H_1_R knockout animal studies were employed to explore the molecular mechanisms driving the sleep-promoting effects. Sleep latency reduction and increased sleep duration in the pentobarbital-induced sleep test in mice were observed with curcuminoids and their constituents. Additionally, curcuminoids notably prolonged the duration of non-rapid eye movement sleep (NREMS) and decreased sleep latency while not affecting rapid eye movement sleep (REMS) and delta activity. Molecular modeling indicated the interaction of curcumin, demethoxycurcumin, and bisdemethoxycurcumin (Figure 2) with H_1_R. Binding affinity assays revealed significant H_1_R binding of both curcuminoids and their constituents, with a *K*_i_ value of 1.49 μg/mL. Furthermore, the administration of curcuminoids reduced sleep latency and increased NREMS frequency in wild-type mice, but this effect was absent in H_1_R knockout mice [19]. Thus, curcuminoids and their constituents from *C. longa* roots reduced sleep latency and increased sleep duration by enhancing the quantity of non-rapid eye movement sleep (NREMS) by acting as modulators of histamine H_1_ receptor in the pentobarbital-induced sleep test in mice, indicating their usefulness in treating insomnia [19,20].

#### 2.1.3. *Dodonaea viscosa* Jacq. (Hawaiian Name: ‘A‘ali‘i)

**Figure 3:**
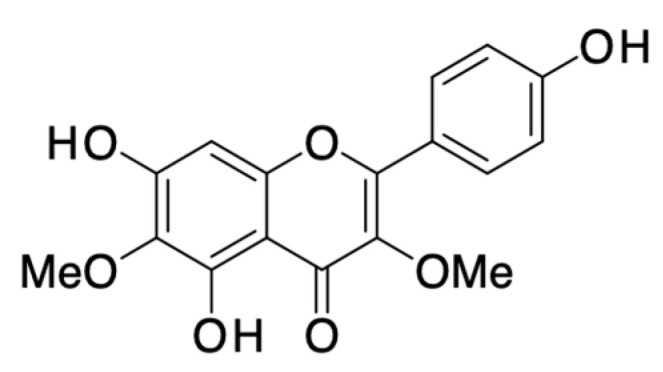
Viscosine from *D. Viscosa*.

*Dodonaea viscosa,* or hopbush, is a flowering plant belonging to the Sapindaceae family. In 2015, Karim et al. investigated the impact of the viscosine (Figure 3) on GABA-evoked currents at recombinant GABAA receptors and subsequently explored its effects on anxiety, sedation, and anticonvulsant activities. Viscosine, within the concentration range of 1 to 300 μM, demonstrated a positive modulation of GABA-evoked currents in human α1β2γ2L and α2β2γ2L GABAA receptors expressed in Xenopus oocytes. Notably, this modulation was insensitive to flumazenil. Behavioral assessments revealed that viscosine administration at doses between 10 and 100 mg/kg (intraperitoneal) produced significant anxiolytic effects, as evidenced by the elevated plus maze, light–dark, and open-field tests. Moreover, higher doses (100 mg/kg intraperitoneal) induced sedative effects, as observed in the hole board and thiopental-induced sleep time tests. Notably, the anxiolytic effect observed in the elevated plus maze test was unaltered by flumazenil, while pentylenetetrazole (PTZ) completely counteracted the effect. This indicates that the anxiolytic activity was mediated through non-benzodiazepine sites on GABAA receptors. Furthermore, viscosine exhibited dose-dependent anticonvulsant effects at doses of 10 to 100 mg/kg (intraperitoneal) in PTZ, picrotoxin, and bicuculline-induced seizure paradigms. In summary, the findings from this study suggest that the anxiolytic and anticonvulsant properties of viscosine likely stem from its positive allosteric modulation of GABA at distinct GABAA receptor subtypes. This study summarized that viscosine (Figure 3) isolated from *D. Viscosa* modulates GABAa receptors at a site different from the classical benzodiazepine site. It showed anxiolytic effects at low doses and sedative effects at high doses. Viscosine also has anticonvulsant effects as measured by various convulsion-inducing agents. These findings suggested that viscosine may be a promising candidate for the development of new GABAa receptor subtype-selective drugs for treating anxiety and seizures [21].

#### 2.1.4. *Mimosa pudica* L. 

**Figure 4:**
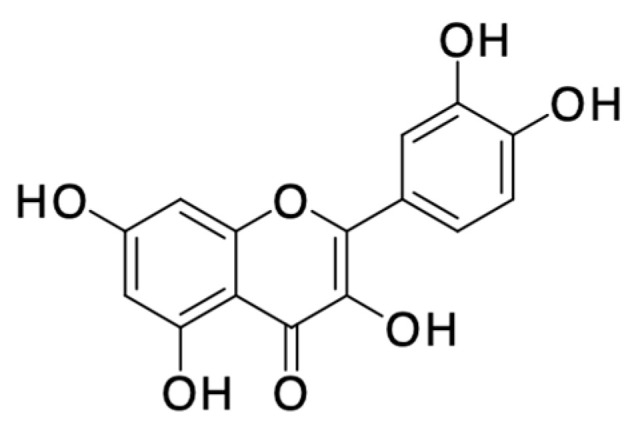
Quercetin from *M. pudica*.

*Mimosa pudica* has been suggested to possess anxiolytic effects, likely due to its antagonistic effect on the serotonin (5-HT) receptor. Furthermore, its antidepressant and memory-enhancing effects have been suggested, although the exact mechanism has yet to be determined. Additionally, *M. pudica* has been indicated to have a therapeutic effect by suppressing α-synuclein and dopaminergic neurodegeneration in vitro (human neuroblastoma SH-SY5Y cells), treated with 300 μg of *M. pudica* extract likely due to the antioxidative activity of its constituent quercetin (Figure 4), as well as its ability to regulate neuroactive ligand–receptor interactions and serotonin and dopamine synapses, as Liaqat et al. reported in 2022 [22]. In 2022, Nafilah et al. conducted an evaluation of the sedative effects of a dry extract of *M. pudica* leaves in powder. This research involved 15 male white mice divided into five distinct treatment groups, including a positive control (diazepam), a negative control (instant powder base), and various doses of dried *M. pudica* leaf extract. The mice underwent treatment and were assessed on a rotarod test at 30 min intervals from 30 to 150 min, measuring the average frequency of falls. The results highlight that the 600 mg/KgBB dose of the *M. pudica* leaf extract demonstrated sedative efficacy comparable to the positive control. Thus, this study demonstrated the sedative properties of the dried *M. pudica* leaf extract in male white mice, with the 600 mg/KgBB dose showing a level of effectiveness similar to the positive control. These findings underscore the potential of *M. pudica* extract as a natural sedative agent [23].

#### 2.1.5. *Piper methysticum* G. Forst. (Hawaiian Name: Awa/Kava)

*Piper methysticum*, commonly known as kava or kava-kava, is a common plant in the Pacific islands belonging to the Piperaceae family. In 2019, Kuchta et al. published their study to confirm the effectiveness and safety of *P. methysticum* in the short-term treatment of nervous anxiety, tension, and restlessness, particularly in acute cases. A total of 156 patients (86 female and 70 male) had undergone treatment (67–125 mg of semiliquid extract of *P. methysticum* extract), and their progress had been meticulously recorded. The findings unveiled a median treatment duration of 28 days, during which all individual symptoms exhibited noteworthy and clinically significant enhancements. The most significant improvements were seen in the symptoms of nervous tension and restlessness, with the safety of the treatment being found to be excellent. These results could apply to related phobias, nervous tension, and restlessness, according to the International Classification of Diseases (ICD-10) [24]. 

Then, Feizi et al. discussed a clinical trial that found that non-psychotic anxiety sleep disorders could be treated effectively and safely with an extract of *P. methysticum* [25]. The exploration of *P. methysticum* extract affects sleep in sleep-disturbed rats compared to flunitrazepam. Electrodes were implanted to monitor brain and muscle activity. *P. methysticum* extract (300 mg/kg) reduced sleep onset time, while flunitrazepam shortened sleep onset, decreased wake time, and increased non-REM sleep. Flumazenil blocked flunitrazepam’s effects but not *P. methysticum* extracts. *P. methysticum* extract increased delta activity during non-REM sleep, improving sleep quality, while flunitrazepam reduced delta power. Flumazenil had no significant impact on *P. methysticum* extract or flunitrazepam effects. *P. methysticum* extract acts as an herbal sleep aid and enhances sleep quality. In addition, the efficacy and safety of *P. methysticum* extract for sleep issues linked to anxiety. In a 4-week, double-blind trial with 61 participants, those taking *P. methysticum* extract (doses of 200 mg) showed significant improvements in sleep quality and recuperation compared to baseline (*p* = 0.007 and *p* = 0.018, respectively). Additionally, *P. methysticum* extract displayed superior effects in reducing psychic anxiety (*p* = 0.002) and enhancing overall well-being, as indicated by self-ratings and clinical evaluations. The extract was well-tolerated, with no adverse events or notable changes in clinical parameters. This highlights the potential as a safe and effective option for managing sleep disturbances associated with non-psychotic anxiety disorders. Furthermore, in a pilot study, 24 patients with stress-related insomnia were treated with *P. methysticum* extract (120 mg daily) for 6 weeks, followed by 2 weeks off. Then, 19 patients received valerian (600 mg daily) for another 6 weeks. Both *P. methysticum* extract and valerian significantly reduced stress (*p* < 0.01) and improved insomnia (*p* < 0.01). Side effects were minimal, with around 58% of patients experiencing none. The most common side effect was vivid dreams with valerian (16%) and dizziness with *P. methysticum* extract (12%). More research is needed to understand the roles of these herbal remedies in treating stress and insomnia [25].

**Figure 5:**
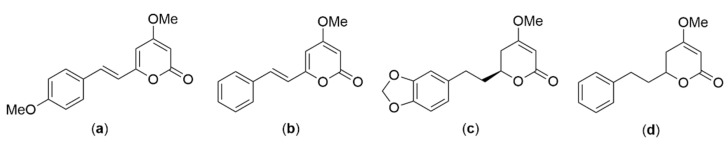
Kavalactone derivatives from *P. methysticum*: (**a**) (*E*)-yangonin, (**b**) (*E*)-desmethoxyyangonin, (**c**) (*S*)-dihydromethysticin, and (**d**) 7,8-dihydrokavain.

In 2020, Volgin et al. summarized that *P. methysticum* extract could promote barbiturate-evoked sleep-in synergy with other sedative herbs such as *passiflora* and alter the melatonin pathway. Its sedative effects are associated with increased binding of GABA receptors in various brain regions. Moreover, these reports indicate that oral consumption of dihydrokavain and dihydromethysticin (Figure 5) has been linked to an improvement in sleep quality in rodents [26]. 

Later, Bian et al. revealed that *P. methysticum* has been used for several decades in the U.S. as a dietary supplement to relieve stress, improve sleep and memory, and regulate mood. The recommended daily dose for adults is 60–250 mg kavalactones (Figure 5), taken for one to two months. *P. methysticum* alcohol tinctures are also available as dietary supplements, but this is potentially dangerous due to the interaction between *P. methysticum* and ethanol. Capsules have also been filled with *P. methysticum* powder to be used as dietary supplements, though the safety of this practice is yet to be determined [27].

In 2022, Sealy et al. reviewed that the active pharmacological components present in *P. methysticum* are collectively referred to as kavalactones (Figure 5). *P. methysticum* acts to enhance GABA transmission, whereby at low micromolar concentrations, *P. methysticum* extracts augment the binding of molecules to the GABA-A receptor. This augmentation strengthens the binding of GABA itself and promotes the influx of chloride ions. Importantly, kavalactones (Figure 5) do not influence the binding of flunitrazepam, indicating that their impact on GABA-A receptors does not involve the benzodiazepine receptor. Although *P. methysticum* holds promise in improving sleep, only two formal studies have explored its effects. In one study, daily administration of kava over six weeks was shown to alleviate sleep disturbances induced by stress. Another study revealed that kava extract has the potential to enhance sleep quality and the restorative aspects of sleep-in individuals with anxiety-related sleep issues. Notably, *P. methysticum* has received approval from the German Commission E for addressing anxiety and sleep problems [28].

In 2023, Pont-Fernandez et al. mentioned that *P. methysticum* is frequently conceptualized as an anxiolytic agent with minimal risks or adverse effects on Reddit. It is important to evaluate the potential risks, benefits, and drug–drug interactions of *P. methysticum* due to its increasing usage, particularly among individuals who use other drugs with a potential for misuse or addiction. To do this, it would be essential to conduct probability sampling and clinical practice studies to gain a better understanding of *P. methysticum*’s anxiolytic properties and its potential side effects [29].

#### 2.1.6. *Stachytarpheta cayennensis* (Rich.) Vahl (Introduced to Hawaiian; Hawaiian Name: Ōi or Ōwī)

*Stachytarpheta cayennensis* is a species of flowering plant in the Verbenaceae family and is known as blue snakeweed. In 2013, Olayiwola et al. reported the investigation of the scientific basis for the ethnomedicinal use of *S. cayennensis* leaves in managing insomnia and anxiety. The sedative and anxiolytic effects of various extracts (methanol, ethyl acetate, butanol, and aqueous) were examined in mice. In mice, sedation was evaluated by measuring reduced novelty-induced rearing (NIR) and spontaneous locomotor activity (SLA), as well as increased sleeping time induced by pentobarbitone (PIST). An elevated plus maze was used to assess the anti-anxiety effects (methanol 2.5–5.0 mg/kg, butanol 5.0 mg/kg, aqueous 20.0 mg/kg, ethylacetate 25.0 mg/kg, intraperitoneal administration; i.p.). The LD_50_ was calculated for the extract and its fractions after intraperitoneal administration using the Locke method. The methanolic extract, butanol, and aqueous fractions reduced rearing and locomotion while prolonging pentobarbitone-induced sleep. In contrast, the ethylacetate fraction increased rearing and locomotion while reducing pentobarbitone-induced sleep. The butanol and aqueous fractions, not the methanol extract, exhibited anti-anxiety effects through open-arm avoidance. The administration of naltrexone (2.5 mg/kg, i.p.) reversed the effects of the aqueous fraction on rearing, locomotion, and pentobarbitone sleep. Flumazenil (2.0 mg/kg, i.p.) nullified the effects of both the methanolic extract and the butanol fraction on rearing, locomotion, pentobarbitone sleep, and anxiety models. The methanolic extract, butanol, and aqueous fractions demonstrated sedative activity, while the ethylacetate fraction had a stimulant effect. Anxiolytic effects were observed in the aqueous and butanol fractions but not in the main methanol extract or ethylacetate fraction. The involvement of GABA receptors in sedative and anxiolytic activities was suggested by the blocking effect of flumazenil on rearing, locomotion, and the elevated plus maze. Additionally, opioid receptors were found to be implicated in the sedative activity. This study provided scientific validation for the traditional use of *S. cayennensis* leaves in managing insomnia and anxiety, shedding light on their mechanisms of action. Overall, this study scientifically supports the traditional use of *S. cayennensis* leaves for managing insomnia and anxiety, providing valuable insights into the neural mechanisms underlying their medicinal properties [30].

**Table 1:** Hawaiian plants with beneficial effects on sleep.

Scientific Name	Part of Used	Effective Extract/Chemical	Properties	References
*Acacia koa*	NA *	NA *	Traditional used as sleep-inducing properties	[16]
*Curcuma longa*	NA *	Curcumin	Chronic sleep deprivation (CSD) effect in rat models	[17]
	Rhizomes	Extracts	Anxiolytic and hypnotic effects through the interaction with GABAergic and nitrergic systems in mice	[18]
	Roots	Curcuminoids/ Extracts	Reduction in sleep latency and enhancement of NREMS via H_1_R blockade in mice	[19,20]
*Dodonaea viscosa*	NA *	Viscosine	Sedative and anxiolytic effects	[21]
*Mimosa pudica*	Entire plant	Quercetin	Antagonistic effect on the serotonin (5-HT) receptor in vitro	[22]
	Leaves	Extracts	Sedative effects in mice	[23]
*Piper methysticum*	Rhizomes	Ethanol extracts	Treatment of nervous anxiety, tension, and restlessness in acute cases	[24]
	Rhizomes/ Roots	Extracts	Effective and safe treatment for non-psychotic anxiety sleep disorders in clinical trial	[25]
	NA *	Extracts	Promote barbiturate-evoked sleep-in synergy	[26]
	NA *	Kavalactones/ Powder	Dietary supplements to relieve stress, improve sleep and memory, and regulate mood	[27]
	NA *	Kavalactones	Facilitate GABA transmission, shows potential for improving sleep	[28]
	NA *	NA *	Anxiolytic with few risks or adverse effects	[29]
*Stachytarpheta cayennensis*	Leaves	Extracts	Sedative and anxiolytic effects	[30]

* NA: Not available.

### 2.2. Hawaiian Plants with Beneficial Effects on Anxiety (Table 2)

#### 2.2.1. *Azadirachta indica* A. Juss. (Indian Lilac, Hawaiian Name: Neem)

*Azadirachta indica* belonging to the Meliaceae family, is known as, or commonly referred to as neem, nim tree, or Indian lilac. It has been widely distributed in tropical and subtropical regions. Anxiety may occur as a symptom of clinical major depression. In 2019, Falaki et al. examined the anxiolytic effects of the leaf extract of *Tapinanthus globiferus* grown on *A. indica* and found that it had a significant anxiolytic activity. This finding supported the traditional use of this plant for anxiety relief and was consistent with the outcomes of previous studies that showed antidepressant activity in methanol extracts of the same plant and anxiolytic and antidepressant activity in a crude aqueous extract from the stem bark of another specie, *T. dodoneifolius* [31].

In 2022, Hawiset et al. investigated the potential anxiolytic and antidepressant-like effects of an aqueous extract obtained from the flowers of *A. indica* in rats subjected to stress. Male Wistar rats were divided randomly into two main groups: a control group and a stress-induced group. Rats in the stress group were exposed to a 3-h restraint stress. Over a duration of 30 days, stressed rats were administered different substances: a control solution, diazepam, fluoxetine, and *A. indica* extract at doses of 250, 500, and 1000 mg/kg body weight. To assess anxiolytic and antidepressant-like behaviors, the researchers employed three behavioral tests: the elevated plus-maze test (EPMT), the forced swimming test (FST), and the open field test (OFT). The EPMT involved measuring the percentage of open-arm entries and the time spent in open arms. Notably, stressed rats treated with diazepam or *A. indica* flower extract at a dosage of 500 mg/kg BW exhibited significant improvements in these measurements. Moreover, rats exposed to stress and subsequently administered fluoxetine or *A. indica* flower extract at all tested doses displayed a noteworthy reduction in immobility time during the FST, indicating potential antidepressant-like effects. Notably, no notable variance in spontaneous locomotor activity was observed across any of the experimental groups.

Further analysis revealed that rats subjected to stress and treated with either standard positive control drugs or *A. indica* flower extract showed elevated levels of brain dopamine (DA) and serotonin (5-HT), accompanied by reduced blood cortisol levels compared to stressed rats given the control solution. Additionally, administration of *A. indica* flower extract did not result in any harmful effects on the rats’ liver tissue, as determined through examination. This study highlighted that *A. indica* flower extract, particularly at a dose of 500 mg/kg BW, could potentially have anxiolytic and antidepressant-like effects in stressed rats, as demonstrated through behavioral tests and biochemical analyses of neurotransmitter levels. Furthermore, the extract showed no detrimental impact on the rats’ liver tissue. Administration of the extract could attenuate the stress-induced behavioral impairment by regulating dopaminergic and serotonergic functions in the rats, indicating potential anxiolytic and antidepressant applications of the extract [32].

#### 2.2.2. *Cocos nucifera* L. (Coconut, Hawaiian Name: Niu)

*Cocos nucifera* (coconut) is a member of the palm tree family (Arecaceae) and provides a variety of resources, such as food, fuel, cosmetics, medicine and building materials, to many people in tropical and subtropical regions. The mature seeds of the coconut trees have edible inner flesh and coconut milk, which are commonly included in the diets of those living in these regions. In 2019, Hugar et al. investigated the anti-anxiety effects of an ethanolic extract of *C. nucifera* endocarp in mice using three validated models: the elevated plus maze (EPM) test, open field apparatus (OFT) test, and light–dark transition (LDT) test. In the experimental animal model, the administration of test extracts at doses of 250 and 500 mg/kg resulted in a significant augmentation of both the number of entries and the duration of time spent in the open arms during the elevated plus maze (EPM) test. Moreover, during the open field apparatus test (OFT), the extract derived from the endocarp of the *C. nucifera* demonstrated a significant increase in behaviors such as rearings and crossings across the central area of the square. Furthermore, in the light–dark transition (LDT) test, the *C. nucifera* extract led to a notable increase in the duration spent within the illuminated chamber, an elevation in the number of crossings, and a reduction in the overall duration of immobility. Based on the findings of the investigation, it can be inferred that the ethanolic extract derived from the endocarp of *C. nucifera*, particularly at the doses of 250 and 500 mg/kg, exhibits pronounced anti-anxiety effects. These findings suggested that *C. nucifera* endocarp may be a potential therapeutic agent for anxiety [33].

**Figure 6:**
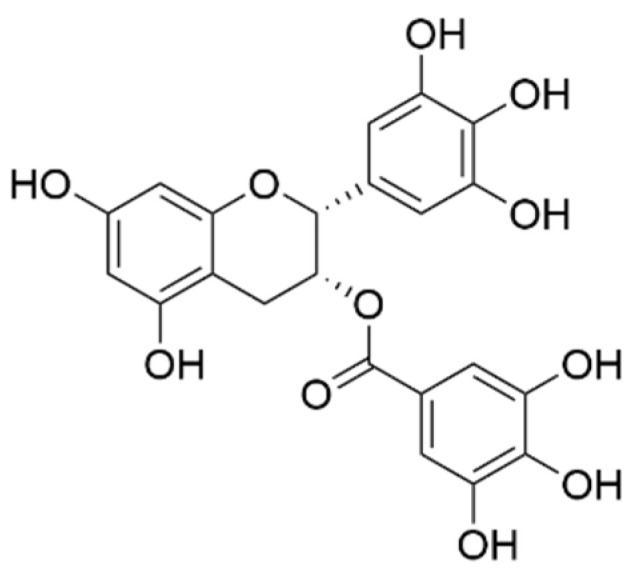
Epigallocatechin gallate (EGCG).

In 2020, Platero et al. evaluated that the combination of coconut oil and epigallocatechin gallate (EGCG, Figure 6) influences IL-6 levels, anxiety, and functional disability in individuals with multiple sclerosis (MS). The research involved a four-month pilot study with 51 MS patients, who were randomly split into two groups: an intervention group and a control group. The intervention group received a daily dose of 800 mg of EGCG and 60 mL of coconut oil, while the control group received a placebo. Both groups followed the same isocaloric Mediterranean diet throughout the study. To assess the outcomes, measures of state and trait anxiety were recorded using the State-Trait Anxiety Inventory (STAI) before and after the study. Additionally, IL-6 levels in the blood were quantified using the ELISA technique, and functional capacity was evaluated using the Expanded Disability Status Scale (EDSS) and the body mass index (BMI). The results indicated a reduction in state anxiety and functional disability among participants in the intervention group. Furthermore, IL-6 levels decreased in both the intervention and control groups. In conclusion, the combined administration of EGCG and coconut oil led to improvements in state anxiety and functional capacity in MS patients. The decrease in IL-6 levels might be attributed to the antioxidant properties of the Mediterranean diet, along with potential benefits related to improved BMI. These results suggested that EGCG and coconut oil may be beneficial in improving the symptoms of multiple sclerosis (MS), likely due to the antioxidant properties of the Mediterranean diet and its influence on improving body mass index (BMI) [34].

#### 2.2.3. *Curcuma longa* L. (Turmeric, Indian Saffron, Hawaiian Name: Ōlena)

In a 2020 review, Ramaholimihaso et al. reported that curcumin (Figure 2) from *C. longa* has been found to possess anti-inflammatory and antioxidant properties that may provide therapeutic benefits in the treatment of depression. A patient with depression often experiences a lot of anxiety. Curcumin has demonstrated promising effects in reversing depressive-like behaviors induced by chronic stress in mice. It appears to enhance serotoninergic and dopaminergic transmission while inhibiting the activity of the monoamine oxidase (MAO-A) enzyme. Observation of similar outcomes in rats found that curcumin increased serotonin and dopamine levels in a dose-dependent manner and inhibited monoamine oxidase enzymes. Recent investigations also revealed that curcumin elevated norepinephrine, serotonin, and dopamine levels in the frontal cortex, hippocampus, and striatum of rats. In a study of curcumin’s effects on serotonin (5-HT) receptors, an antidepressant-like effect was linked to interactions with serotonin receptors 5-HT1A/1B and 5-HT2C. Correspondingly, the antidepressant effect of curcumin was associated with increased expression of 5-HT1A receptors, supported by elevated 5-HT1A receptor mRNA levels across various hippocampal subfields after curcumin administration. These findings noted increased 5-HT1A receptor expression following curcumin administration in chronically stressed mice. Notably, the antidepressant-like effect was countered when a 5-HT1B receptor antagonist was administered. Collectively, these animal studies provide robust evidence that curcumin has the potential to modulate monoaminergic systems in preclinical rodent models, offering insights into its antidepressant properties. It has been suggested that curcumin may be used as a prophylactic agent due to its neuroprotective effects against stress-induced toxicity. Nevertheless, more clinical trials are needed to determine its efficacy and optimal dosage [35]. 

In addition, in 2019, Bhat et al. reviewed that curcumin (Figure 2) has demonstrated potent antidepressant effects in animal models of depression. Its mechanism involves the inhibition of monoamine oxidase (MAO-A and MAO-B) enzymes, leading to elevated levels of norepinephrine, serotonin, and dopamine. By modulating extracellular regulated kinase (ERK) activity, curcumin enhances the expression of brain-derived neurotrophic factor (BDNF) in the amygdala of mice, contributing to its antidepressant actions. Additionally, curcumin has been found to stimulate hippocampal neurogenesis and elevate BDNF levels in a mouse model of chronic stress. Its anxiolytic-like effects are thought to stem from the reduction in pro-inflammatory mediators like iNOS and COX-2 mRNA, achieved through the NF-κB signaling pathway. The inhibition of proinflammatory cytokine IL-1β through the NF-κB pathway further confirms curcumin’s anti-inflammatory potential in the context of depression. Thus, curcumin promoted hippocampal neurogenesis, improved brain-derived neurotrophic factor (BDNF) levels in a mouse model of chronic stress and exerted anxiolytic effects via decreased levels of pro-inflammatory mediators and inhibition of proinflammatory cytokines IL-1β via NF-κB pathway [36]. 

A random effects meta-analysis involving five studies with a total of 284 participants (160 in the curcumin group and 124 in the comparison group) revealed a significant and substantial positive effect of curcumin on anxiety symptoms. The effect size was large, indicated by Hedge’s g value of −2.62, with a 95% confidence interval ranging from −4.06 to −1.17 (*p* < 0.001). The level of variability among the studies (heterogeneity) was low, with an *I*² value of 23.89%. Further analysis, by systematically excluding each individual study from the overall effect size, did not yield any statistically significant differences in the results. This finding contrasts with a recent rat model study, which reported no anxiolytic effects or changes in behavioral despair associated with curcumin. Additionally, this earlier research did not identify any interactions between curcumin and the benzodiazepine site of the γ-aminobutyric acid (GABA)-A receptor.

Nonetheless, various potential mechanisms could explain the observed anxiety-reducing effects of curcumin. For instance, curcumin may enhance serotoninergic transmission in rats by inhibiting monoamine oxidase (MAO), leading to increased serotonin levels in the brain’s medial prefrontal cortex. Other studies suggested that curcumin promotes the conversion of hepatic α-linoleic acid into docosahexaenoic acid (DHA), an omega-3 fatty acid with anxiolytic-like properties and enhances its accumulation in the brain. Additionally, curcumin has been found to suppress the synthesis of a specific form of nitric oxide synthase (iNOS), which tends to increase during stress in the brain cortex, thereby producing effects akin to anxiolysis. It is worth noting that these mechanistic insights are derived solely from preclinical studies, and as of now, no dedicated trials assessing the efficacy of curcumin for anxiety disorders in humans have been conducted, as suggested by Fusar-Poli et al. [37]. 

In 2021, Moragrega et al. described that curcumin, an active compound found in *C. longa*, exhibits antidepressant effects through intricate mechanisms involving both the serotonergic system and the AC-cAMP pathway. Additionally, curcumin enhances the antidepressant actions of subliminal doses of drugs like fluoxetine, venlafaxine, and bupropion. When combined with piperine, a bioavailability enhancer, curcumin’s impact is further amplified. This combination not only boosts the neurotransmitter serotonin (5-HT) systems mediate dopamine (DA) but also intensifies the inhibitory effects on MAO-A, surpassing the effects of curcumin administered alone. In studies conducted on rats subjected to bilateral olfactory bulbectomy, a procedure that induces depression-like behavioral changes, curcumin effectively counteracts these alterations, similar to conventional antidepressants. This correction is attributed to curcumin’s ability to mitigate immobility time and reverse the behavioral anomalies triggered by bulbectomy. Moreover, curcumin demonstrates a protective effect on rat hippocampal neurons against damage caused by chronic stress. This safeguarding mechanism involves the positive regulation of receptors like serotonin 1A (5-HT1A) and brain-derived neurotrophic factor (BDNF) expression, both crucial for hippocampal neurogenesis. Exploration of curcumin’s anxiolytic and antidepressant-like properties through various selective tests. Their findings dismiss the idea that curcumin’s effects are tied to the benzodiazepine site on the GABAA receptor. Instead, these effects likely arise from interactions with other receptor subunits or central nervous system neurotransmitter systems, corroborating the previously mentioned mechanisms underlying curcumin’s antidepressant actions. This study examined the anxiolytic and antidepressant-like effects of curcumin in various tests and demonstrated that its effects were not likely due to interaction with the GABAa receptor benzodiazepine site, suggesting that curcumin may modulate other receptor subunits or interact with other central nervous system neurotransmitter systems [38]. 

In 2021, Latif et al. conducted a study to assess the impact of consuming natural turmeric on cardiovascular risk factors, mental well-being, and serum homocysteine levels in overweight and obese women. The study utilized a pre-post, single-arm design carried out at the Department of Physiology in Imam Abdulrahman Bin Faisal University, located in Dammam, Saudi Arabia. The study included 36 female university students, all of whom had a body mass index (BMI) of 23 kg/m² or higher. Over a span of 90 days, participants were provided with a daily dosage of 2 g of *C. longa* in the form of capsules. The study collected initial and post-intervention data on various aspects, including anthropometric measurements, blood pressure, serum homocysteine levels, and indicators of mental health such as stress, anxiety, and depression scores.

Additionally, the potential confounding variables of dietary intake and physical activity were also assessed. The results indicated noteworthy reductions in multiple anthropometric measurements after the intervention, encompassing body weight, body mass index, waist circumference, hip circumference, body fat percentage, and systolic blood pressure. There was also a significant decrease in anxiety scores. However, there were no significant alterations observed in homocysteine levels, stress, or depression scores. Dietary intake and physical activity remained relatively constant during the study duration. Thus, this study demonstrated that daily consumption of 2 g of *C. longa* over 90 days could potentially lead to weight loss, lowered body fat percentage, reduced systolic blood pressure, and decreased anxiety levels in young, overweight and obese women.

Nonetheless, there were no significant changes in homocysteine levels, stress, or depression scores throughout the study period. The effects of *C. longa* supplementation (2 g/d for 90 days) on obesity-related and cardiovascular-disease risk factors in overweight or obese females. Results showed that *C. longa* was able to reduce weight, body fat percentage, systolic blood pressure, and anxiety levels in the participants. These findings suggested that *C. longa* may be a promising treatment for obesity-related and cardiovascular disease risk factors besides anxiety in this population [39]. 

Current evidence suggests that curcumin (Figure 2) may have antidepressant properties, as evidenced by its ability to improve depressive and anxiety behavior, increase monoamines and brain-derived neurotrophic factor levels, inhibit pro-inflammatory cytokines, and reduce neuronal apoptosis. Additionally, curcumin has been reported to improve insulin sensitivity, reduce cortisol levels, and reverse metabolic abnormalities. Further research is needed to evaluate the efficacy of curcumin in depression treatment, as the dose and formulation of curcumin vary, as described and reviewed by Matias et al. in 2021 [40]. Later, Lopresti et al. studied the impact of a curcumin extract called Curcugen™ on gastrointestinal symptoms, mood, and overall quality of life in adults who reported digestive issues. Additionally, the study aimed to uncover potential mechanisms of curcumin’s effects by investigating its impact on gut microbiota and small intestinal bacterial overgrowth (SIBO). The research was conducted over 8 weeks, employing a double-blind, randomized controlled trial involving 79 adults with self-reported digestive complaints. Participants were randomly assigned to receive either a placebo or 500 mg of the curcumin extract, Curcugen™. Multiple measurements were taken, including the Gastrointestinal Symptom Rating Scale (GSRS), Depression, Anxiety, and Stress Scale-21 (DASS-21), Short Form-36 (SF-36), as well as a SIBO breath test. The study also analyzed the participants’ intestinal microbial profiles using 16S rRNA sequencing. The results, derived from self-reported data of 77 participants, demonstrated that those who received curcumin experienced a notable reduction in the total GSRS score, indicating an improvement in gastrointestinal symptoms when compared to the placebo group. Furthermore, there was a significant decrease in the anxiety score measured by DASS-21 in the curcumin group. However, no other substantial differences were observed in self-reported data between the two groups. Notably, curcumin did not seem to have a significant impact on intestinal microbial composition or SIBO test results. Importantly, curcumin was well-tolerated without any significant adverse events. To conclude, administering the Curcugen™ curcumin extract at a dosage of 500 mg once daily over an 8-week period led to greater enhancements in both digestive complaints and anxiety levels among adults reporting such issues. While the study did not identify significant changes in gut microbiota or SIBO when compared to the placebo, further research using larger participant samples and more comprehensive microbial analysis methods will be crucial. Exploring additional potential mechanisms underlying curcumin’s beneficial effects on gastrointestinal symptoms, such as its influence on factors like intestinal barrier function, inflammation, neurotransmitter activity, and visceral sensitivity, also warrants investigation. Further research with larger sample sizes and methods that allow for more detailed microbial analyses is needed to further explore the potential benefits of curcumin on gastrointestinal health and anxiety. Additionally, future studies should focus on other potential mechanisms associated with curcumin’s gastrointestinal-relieving effects, such as its influence on intestinal barrier function, inflammation, neurotransmitter activity, and visceral sensitivity [41]. 

Norwitz et al. reviewed that curcumin has been investigated as a possible remedy for various brain-related conditions such as Alzheimer’s disease, Parkinson’s disease, depression, anxiety, and related conditions. The mechanisms of curcumin modulation of neurotransmitters like dopamine, serotonin, and cortisol, and regulation of microRNAs and histone deacetylases (HDACs). Preclinical assessments of curcumin’s impact on anxiety in animal models have reinforced its potential as an anti-anxiety agent. In studies involving rodents exposed to anxiety-inducing factors, curcumin intervention effectively mitigated anxiety-related behaviors. These findings showed that curcumin not only significantly reduced anxiety-like behaviors but also positively influenced neurotransmitter and hormone levels. Several rigorously conducted trials involving human participants, characterized by randomization, double-blinding, and placebo controls, have substantiated the anxiety-reducing effects of curcumin supplementation. For instance, in patients with diabetes, an eight-week curcumin supplementation regimen led to decreased anxiety. Likewise, a crossover trial involving obese individuals demonstrated that curcumin supplementation over a 30-day period resulted in lowered anxiety scores. A meta-analysis encompassing five studies further revealed an overall significant reduction in anxiety due to curcumin supplementation, with a substantial effect size (Hedge’s g = −2.62). However, it is important to acknowledge the limitations inherent in curcumin research. Critics have raised concerns about the potential overhyping of the health advantages associated with turmeric and its active constituents. A comprehensive analysis highlighted the chemical instability of curcumin and its potential to interfere with assay results, suggesting that positive findings in experimental models might be skewed.

Furthermore, variations in supplement purity and formulations among different studies have hindered reproducibility. Curcuminoids, being fat-soluble, exhibit limited bioavailability when administered alone or in aqueous solutions. To enhance bioavailability, curcuminoids should be consumed with fats, and approaches such as liposomes and nanoparticles are being explored for their administration. Notably, the positive curcumin-anxiety studies mentioned earlier utilized techniques to boost curcumin bioavailability, including nano-curcumin and co-administration of bioperine, which amplifies curcumin absorption by 20-fold. As such, future research efforts should concentrate on investigating these more bioavailable forms of curcumin, as well as examining curcumin’s impact on the human microbiome, a pathway not reliant on systemic absorption. The connection between *C. longa* and the previous section on omega-3 fatty acids is noteworthy. Humans struggle to efficiently convert alpha-linolenic acid (ALA) into eicosapentaenoic acid (EPA) and docosahexaenoic acid (DHA). Curcumin could enhance this conversion by elevating levels of the enzymes responsible for DHA synthesis. This not only heightens DHA levels in the brain but also holds implications for anxiety regulation. Rodents treated with a combination of ALA and curcumin displayed reduced anxiety [42]. 

In 2022, Lopresti et al. suggested that disturbances in the functioning of neurotransmitters like serotonin (5-HT), dopamine, noradrenaline, and glutamate have consistently been observed in cases of depression. Animal trials have shown that curcumin, a compound found in *C. longa*, can influence the levels and activity of these neurotransmitters. For instance, when administered to mice, curcumin quickly improved depressive behaviors by positively affecting the 5-HT1A/2A receptor. In mice that had their ovaries removed, curcumin changed depressive behaviors and increased serotonin levels in various brain regions by promoting the expression of key genes involved in serotonin regulation while reducing the activity of a specific enzyme called monoamine oxidase A. In rats subjected to chronic stress, curcumin lessened anxious behaviors and reversed stress-related reductions in serotonin levels in brain areas such as the hippocampus, amygdala, and striatum. Additionally, curcumin had effects on dopamine and noradrenaline levels in different brain regions in rats that had their ovaries removed, counteracted diabetes-induced changes in dopamine receptors, and mitigated dopamine depletion triggered by a pesticide called rotenone. Furthermore, curcumin exhibited the ability to protect against damage caused by excess glutamate, a neurotransmitter linked to neurotoxicity. It achieved this by influencing the activity of *N*-methyl-*D*-aspartate (NMDA) receptors, which are important for glutamate signaling. Curcumin boosted the expression of a specific subunit of NMDA receptors known as GluN2A and encouraged the activation of a specific protein in α-amino-3-hydroxy-5-methyl-4-isoxazolepropionic acid (AMPA) receptors, termed GluR1. These actions collectively indicate that curcumin has the potential to impact various neurotransmitter systems and pathways, offering promise as a potential therapeutic agent for depression and related conditions. There was evidence supporting the efficacy of curcumin as a treatment for depression, as demonstrated by animal and human trials and confirmed by multiple meta-analyses. However, further research is necessary to assess the safety, tolerability, and effectiveness of different curcumin extracts, given their varying levels of oral bioavailability [43].

#### 2.2.4. *Morinda citrifolia* L. (Noni–Indian Mulberry, Great Morinda, Cheese Fruit)

**Figure 7:**
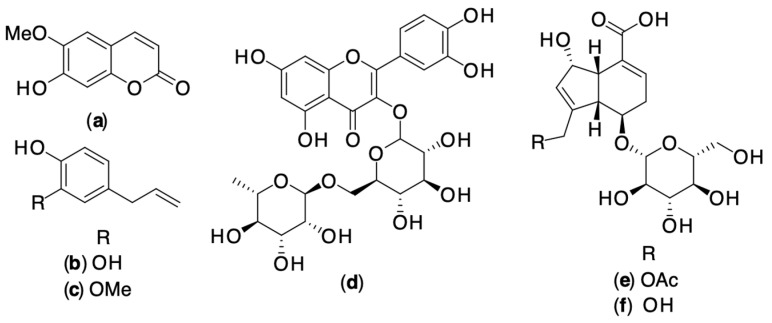
Major compounds from *M. citrifolia*: (**a**) scopoletin, (**b**) hydroxychavicol, (**c**) eugenol, (**d**) rutin, (**e**) asperulosidic acid, and (**f**) deacetylasperulosidic acid.

*Morinda citrifolia* (noni) is a perennial, fruit-bearing tree belonging to the Rubiaceae family, found in Southeast Asia, consumed as both food and medicine for over 2000 years [44,45,46]. In 2019, West et al. reported the HPLC analysis and the effects of *M. citrifolia* fruit puree juice on the two major endocannabinoid degradation enzymes, fatty acid amide hydrolase (FAAH) and monoacylglycerol lipase (MAGL). HPLC analysis unveiled the presence of phytochemical components, including deacetylasperulosidic acid (DAA), asperulosidic acid (AA), scopoletin, rutin, and quercetin (Figure 7) within *M. citrifolia* juice samples. Disruption of fatty acid amide hydrolase (FAAH) activity prevents the effects of chronic stress on anxiety. Deacetylasperulosidic acid (DAA) displayed a moderate inhibition of FAAH activity, reducing it by approximately 28.26% with a standard deviation of 4.61%. It is possible that additional compounds present in *M. citrifolia* juice could have also played a role in influencing the observed activity in our experimental tests. An experiment involving *M. citrifolia* juice and its effects on monoacylglycerol lipase (MAGL) activity in a concentration-dependent manner. MAGL is an enzyme responsible for breaking down 2-arachidonoylglycerol (2-AG). The results revealed that as the concentration of noni juice increased, the activity of MAGL decreased, at the lowest concentration of 2.78 μL/mL of *M. citrifolia* juice, was reduced by approximately 32.88% ± 3.62, at a concentration of 5.56 μL/mL of *M. citrifolia* juice, MAGL activity was reduced by approximately 55.71% ± 4.40%, at the highest concentration of *M. citrifolia* juice tested was 18.52 μL/mL, MAGL activity was completely prevented. Hence, these compounds from *M. citrifolia* juice may contribute to the FAAH and MAGL inhibitory activity, potentially supporting mental health, joint health, relieving discomfort, and modulating the immune system [45]. 

A pilot clinical study involving Tahitian *M. citrifolia* juice also reported improved mental health scores. The underlying biological mechanism for these effects is related to Gamma-aminobutyric acid (GABA), a vital inhibitory neurotransmitter in the central nervous system. GABA binds to specific receptors on both pre- and post-synaptic neurons in the plasma membrane. Among potential targets for various central nervous system disorders, the GABA receptor stands out, particularly in anxiety-related conditions. Extensive clinical experience and well-designed experiments have established the significance of GABA in mediating different anxiety disorders. Furthermore, neurotransmitters like norepinephrine and serotonin also play crucial roles in these processes. Alterations in these neurotransmitters have been observed in association with *M. citrifolia* treatment. Medications that modulate the GABAergic or serotonergic systems or that reduce adrenergic activity are commonly used for treating anxiety disorders. Recent research conducted on rats demonstrated a significant reduction in norepinephrine levels in the amygdala and hippocampus, further supporting the potential benefits of *M. citrifolia* in anxiety management, as reviewed by Ramya et al. in 2021 [46]. In addition, epidemiological studies showed that anxiety disorders are more common among people diagnosed with psychotic disorders compared to the general population. *M. citrifolia* fruits have demonstrated neuropharmacological activities, such as anti-depressant and anti-anxiety effects, in both in vivo and in vitro models. The research aimed to investigate the potential antipsychotic effect of *M. citrifolia* fruits using mouse models of climbing activity. The findings of this study revealed an antidopaminergic effect of *M. citrifolia* in mice, suggesting that *M. citrifolia* may exhibit antipsychotic-like activity and could be a viable candidate for the treatment of mental conditions. Furthermore, to evaluate the potential antipsychotic activity of *M. citrifolia* juice, the researchers administered various doses (5, 10, and 100 mL/kg) equivalent to dried *M. citrifolia* juice powder doses (450, 900, and 1800 mg/kg) to mice. The results indicated that *M. citrifolia* juice significantly reduced amphetamine-induced climbing behavior and methamphetamine-induced stereotypy at these different doses. The *M. citrifolia* extract was orally given in combination with apomorphine and methamphetamine over a period of 21 days, and it demonstrated antipsychotic activity. This study adds to the understanding of the neurochemical basis of schizophrenia and highlights the potential antipsychotic properties of *M. citrifolia* extract. However, further research is warranted to fully explore its mechanism of action and its efficacy compared to standard antipsychotic drugs for human clinical use [46].

In various in vivo studies, a considerable body of research has indicated that *M. citrifolia* holds promise for potential pharmacological applications in treating depression and anxiety. A study delved into the possible role of serotonergic and noradrenergic pathways in the antidepressant effects of *M. citrifolia*. This was explored through interactions with substances like a depletory of adrenergic and dopaminergic compounds (AMPT), a serotonergic depletor (PCPA), and a 5HT1A receptor antagonist in mice. The study investigated the influence of the serotonergic system on the antidepressant-like activity of noni in the tail suspension test (TST), a well-accepted animal model for depression research due to its reliability and sensitivity. In the TST mouse model, the administration of noni extract at doses of 0.5 and 0.75 g/kg significantly reduced the duration of immobility, akin to the effect seen after intraperitoneal administration of the standard antidepressant desipramine (30 mg/kg). The activity of biogenic and dietary amines, regulated by key isoenzymes like monoamine oxidase (MAO-A and MAO-B), plays a crucial role. While MAO-A predominantly oxidizes serotonin (5-HT) and noradrenaline (NA), MAO-B primarily oxidizes phenyl ethylamine (PEA), and both can influence dopamine levels. Tranylcypromine, a non-selective and irreversible inhibitor of both MAO-A and MAO-B, has shown effectiveness in treating severe depression. *M. citrifolia* extract, like tranylcypromine, demonstrated the ability to inhibit both MAO-A and MAO-B enzymes, thus potentially contributing to its antidepressant-like effects in mice and subsequently increasing the levels of biogenic amines like 5-HT and NA. Earlier studies indicated that *M. citrifolia*’s antidepressant-like effects are mediated through its interaction with serotonergic and noradrenergic systems. Building upon this, in vitro investigations were conducted to study the inhibitory effects of noni fruit extract and its identified components on MAO-A and MAO-B enzymes, suggesting their therapeutic potential for antidepressant activity. Additionally, ex vivo studies have revealed that noni fruit contains various chemical constituents such as rutin, scopoletin, and coumarin derivatives (Figure 7). Rutin, with its pharmacological properties encompassing antidepressant, antioxidant, neuroprotective, and antianxiety effects, has shown promise. Moreover, compounds like scopoletin and rutin have demonstrated sequential antidepressant activity, potentially interacting with α1- and α2-adrenoreceptors [47].

The study investigated the anxiolytic and antidepressant-like properties of *M. citrifolia*’s potential mechanisms of action. The effects of *M. citrifolia* were examined through drug interaction studies involving selective benzodiazepine receptor antagonist (flumazenil), GABAA receptor antagonist (bicuculine), and 5HT1A receptor antagonist to understand its interaction with neurotransmitter systems associated with anxiety. The findings indicated that *M. citrifolia* extract (1 g/kg, p.o.) exhibited anxiolytic effects in mouse models, similar to the well-known anxiolytic drug diazepam (1 mg/kg, i.p.). This was observed in various tests, such as the elevated plus maze (EPM) and light–dark transition (LDT) tests. Notably, *M. citrifolia* increased the time spent in the open arms of the EPM, similar to the effects of diazepam. Previous in vitro studies suggested that the methanolic extract of *M. citrifolia* fruit binds preferentially to GABAA receptors, which play a critical role in controlling anxiety. Benzodiazepine-GABAergic neurotransmission was highlighted as vital in anxiety control. Further studies involving noni juice administration to rats for 15 days showed increased exploration of uncomfortable open arms in the EPM. This behavior, akin to benzodiazepine treatment, suggested potential anxiolytic effects. Interestingly, motor activity and rearing behavior remained consistent between treatments. In vitro studies demonstrated that the methanolic extract of noni fruit exhibited 75% binding suppression to GABAA receptors, indicating anxiolytic activity. This extract inhibited the binding of the agonist radioligand (3H) muscimol to GABAA receptors at a concentration of 100 mg/mL, thereby highlighting its GABAergic activity. This receptor binding plays a role in regulating the inhibitory neurotransmitter GABA, contributing to anxiolytic and sedative effects. Neurochemical evaluations indicated modifications in the monoaminergic system in *M. citrifolia*-treated rats compared to controls. The changes included alterations in neurotransmitters like norepinephrine (NE), serotonin (5-HT), 3,4-dihydroxyphenylacetic acid (DOPAC), and homovanillic acid (HVA). *M. citrifolia*’s potential to lower these neurotransmitter levels, particularly 5-HT, suggests anxiolytic properties. The effects on norepinephrine (NE), dopamine (DA), and its intermediates point to reduced anxiety levels. The modulation of different neurotransmitters by *M. citrifolia* appears to be mediated through the GABAergic system. *M. citrifolia*’s GABAA receptor binding affinity further supports its potential to regulate monoaminergic neurons in various brain regions. In vitro findings from an elevated plus maze test highlighted noni juice’s anxiolytic effect mediated in part by alterations in monoaminergic neurotransmitter levels.

Consequently, recent research on *M. citrifolia* suggests potential anxiolytic and antidepressant-like effects. These effects are likely mediated through interactions with the GABAergic and serotonergic systems. *M. citrifolia*’s ability to regulate neurotransmitter levels and its GABAA receptor binding affinity underpins its potential therapeutic applications for anxiety and depression. These findings suggest the potential therapeutic applications of *M. citrifolia* in various neurological and psychiatric conditions, including anxiety, as summarized by Begum et al. in 2022 [47].

#### 2.2.5. *Passiflora edulis* Sims (Passionfruit, Hawaiian Name: Lilikoi)

*Passiflora edulis* (Passifloraceae), commonly known as passion fruit, is popular due to its balanced nutrition, juiciness, and attractive nutritional value. It is widely consumed for its essential benefits for health and is popular among consumers [48,49].

In 2019, Gross et al. summarized that *P. edulis* has been traditionally utilized in Mexico for addressing conditions such as nervousness, anxiety, and depression. Extracts derived from its leaves through percolation have demonstrated a depressant impact on the central nervous system in mice. Both hydroalcoholic and aqueous leaf extracts have exhibited anxiolytic effects in rat experiments conducted using the high cross labyrinth. *P. edulis* is recognized for its potential as an anxiolytic and mild sedative. However, it’s important to note that its consumption might lead to drowsiness, and caution is advised against chronic usage or simultaneous administration with other nervous system sedatives and depressants [50]. 

In 2020, He et al. demonstrated the therapeutic effects of *P. edulis*, with the crude extracts (butanol, methanol, ethanol, hydroethanolic, and aqueous extract) exhibiting anxiolytic-like effects in rat models. When administered orally, the aqueous extract of *P. edulis* (at 50, 100, and 150 mg/kg) showed anxiolytic-like effects in the elevated plus-maze and inhibitory avoidance tests without impairing memory processes. Of greater significance, administration of the aqueous extract of *P. edulis* did not disturb the memory function of rats during their adaptation to an open-field test. Conversely, the habituation of rats was hindered by diazepam, albeit through a basic alteration of the open-field apparatus. The methanol extract derived from the aerial parts of *P. edulis* demonstrated anxiolytic effects in mice when administered orally at a dose of 75 mg/kg, as evidenced by its performance in the elevated plus-maze anxiety model. However, an oral dose of 125 mg/kg did not yield a significant anxiolytic response. Alternatively, higher oral doses of 200 and 300 mg/kg resulted in a mild sedative impact. In a similar vein, prior administration of hydroethanolic extracts at 50, 100, and 150 mg/kg, as well as spray-dried leaf powders at 400 and 800 mg/kg of *P. edulis*, displayed anxiolytic properties in the elevated plus-maze test involving mice. These effects were attributed to the presence of a diverse array of flavonoids and their glycosides, underscoring the potential therapeutic value of these extracts [48]. 

Evaluate the protective effects of hydroalcoholic extract derived from *P. edulis* leaves against anxiety induced by sub-acute immobilization stress in mice. The anxiety induction was achieved through sub-acute immobilization stress, followed by an 11-day treatment period. Subsequently, behavioral assessments were conducted using the elevated plus maze (EPM) and open field (OF) tests, followed by the analysis of biochemical parameters, including malondialdehyde (MDA), glutathione (GSH), superoxidedismutase (SOD), catalase (CAT), GABA, GABA-T, and serotonin (5-HT). The findings demonstrate that the administration of *P. edulis* extract at doses of 100 and 200 mg/kg resulted in a significant increase in the number of entries and time spent in the open arms of the EPM, while decreasing entries and time in the closed arms. In the OF test, the extract led to a significant increase in measures such as lines crossed, passages through the center, and center time. These outcomes suggest that *P. edulis* extract potentially offers protection against anxiety. This effect could be attributed to its ability to combat oxidative stress and counteract hyperexcitability by enhancing GABA’s action. The most effective dose, 100 mg/kg, notably increased GSH activity to 4.44 ± 0.24 µmol/g. In mice treated with this dose, the extract significantly reduced three oxidative stress markers: MDA, catalase, and SOD levels were lowered to 0.22 ± 0.01 µmol/g, 1.05 ± 0.15 mmol H_2_O_2_/min/g, and 19.46 ± 0.00 unit/min/mg, respectively, compared to the negative control. Moreover, animals treated with 100 mg/kg of P. edulis extract exhibited a substantial increase (*p* < 0.001) in GABA and 5-HT levels, reaching 4.62 ± 0.28 µg/g and 31.47 ± 1.58 ng/mL, respectively. The activity of GABA-T was also influenced by the treatment with P. edulis; the GABA-T activity, which was 1.27 ± 0.10 in the negative control, significantly (*p* < 0.001) decreased to 0.37 ± 0.00 in the group treated with the 100 mg/kg dose. Ultimately, the findings of the study revealed the beneficial effects of *P. edulis* extract in mitigating anxiety-like behavior in mice. These effects were linked to the extract’s ability to counteract oxidative stress and enhance GABAergic neurotransmission, thereby suggesting its potential as a natural remedy for anxiety-related conditions, as mentioned by Espoir et al. in 2020 [51]. 

Additionally, the anxiolytic-like effect of *P. edulis* extract was tested in a randomized trial using an anxiety model in rats. The elevated plus-maze (EPM) test, incorporating Diazepam as a positive control, demonstrated that diazepam produced an anxiety-reducing effect in mice. This effect was characterized by a notable increase in the percentage of open-arm entries (OE) and total time spent in the open arms (OT) of the EPM without altering the number of entries into enclosed arms. This outcome was consistent with prior research. In a similar manner, treatment with an ethanol extract (EE) derived from *P. edulis* at doses of 200 and 400 mg/kg resulted in a significant rise in the percentage of open-arm entries and total time spent in the open arms, indicating an anxiety-reducing effect. Interestingly, the group treated with 400 mg/kg of EE showed a decrease in the number of entries into enclosed arms, implying a reduction in motor activity, suggesting that at this higher dose, the EE had more of a sedative-like effect. Additionally, administration of various fractions of *P. edulis* extract, such as chloroform (CF), ethylacetate (EAE), and butanol (BF), at a dose of 200 mg/kg, also led to a noteworthy increase in open arm entries and total time spent in open arms, indicating an anxiolytic effect. However, no significant distinctions were observed among these treatment groups. Notably, BF exhibited the highest activity, followed by EAE and CF. Analysis of the animals’ brain GABA levels revealed that after administering *P. edulis* extract and its fractions, there was a substantial increase in GABA neurotransmitter concentration compared to the negative control. This suggests that the anxiolytic-like effects of *P. edulis* are linked to the GABA-ergic system’s activity. The extracts may act as positive allosteric modulators of GABA, and their effects might be influenced by the presence or absence of endogenous neurotransmitters. The effects of CF and BF were particularly pronounced at an oral dose of 200 mg/kg, followed by the EAE fraction. These findings mirrored the results obtained from the elevated plus-maze model. Although there was no significant difference among the different *P. edulis* treatment groups, it implies that the plant’s anxiolytic effects are likely due to a combination of active constituents, such as flavonoids, rather than a single chemical entity. These results align with Appel’s previous research, which suggested that *P. edulis*’s effects on the central nervous system are mediated by GABA system modulation, including interactions with GABAA and GABAB receptors, as well as effects on GABA uptake. To sum up, the extract was found to increase GABA concentrations in the brain, suggesting that it functioned as a positive allosteric modulator of GABA. Metabolomics analyses did not reveal a correlation between the different metabolites and the observed activities, suggesting that the anxiolytic effect was likely due to an additive or synergistic effect of multiple compounds, as reported by Humer et al. in 2020 [52]. 

#### 2.2.6. *Piper methysticum* G. Forst. (Hawaiian Name: Awa/Kava) 

*Piper methysticum* or kava (Piperaceae) is native to islands of the Pacific Ocean. In 2020, Sarris et al. reported that an aqueous extract of dried *P. methysticum* root, when taken in standardized doses of 120, 240, or 250 mg of kavalactones (Figure 5), could not be used as a psychotropic medication to treat generalized anxiety disorder. In a study comprising 171 participants, an analysis was conducted to examine the impact of *P. methysticum* versus placebo on anxiety reduction in 16 weeks. The results indicated that there was no significant difference in anxiety reduction between the two groups. However, it may be effective as an anxiolytic prior to a potential situational anxiogenic event or as an additional management of non-clinical anxiety and stress [53]. 

Investigation of the potential impact of *P. methysticum* extract on changes in behavior caused by amphetamine (AMPH) and its potential relationship with alterations in monoamine oxidase (MAO) activity. In the experiment, mice were administered either a vehicle or *P. methysticum* extract through gavage, followed by either a vehicle or AMPH through intraperitoneal injection, with a two-hour interval. Behavioral tests (elevated plus maze, open field, stereotyped behavior, social interaction, and Y maze) and biochemical assessments (MAO-A and MAO-B activity in the cortex, hippocampus, and striatum) were conducted sequentially. The findings revealed that *P. methysticum* extract demonstrated anxiolytic effects in the elevated plus maze test, increased locomotor activity in the open field test, and reduced MAO-A activity in the cortex, along with decreased MAO-B activity in the hippocampus. *P. methysticum* extract counteracted the impact of AMPH on stereotyped behavior. When *P. methysticum* extract and AMPH were combined, there was an increase in arm entries in the Y maze test, along with elevated MAO-B activity in the striatum. However, *P. methysticum* extract did not mitigate the hyperlocomotion triggered by AMPH in the open field test. Notably, *P. methysticum* extract did not influence social interaction, both in isolation and in combination with AMPH. In closing, *P. methysticum* extract could prevent the appearance of stereotyped behavior induced by amphetamine (AMPH) in mice, suggesting it might be useful in alleviating psychotic symptoms in patients. Furthermore, the same dose of *P. methysticum* extract that promoted anxiolytic effects was found to also reduce the stereotyped behavior, indicating its potential as a therapeutic for both anxiety and psychotic symptoms, as reported by Krum et al. in 2021 [54]. 

The Committee on Herbal Medicinal Products (HMPC) assessment report on *P. methysticum* has assumed that the true indication of *P. methysticum* is generalized anxiety disorder (GAD according to ICD-10 F41.1). The analysis of data to determine which symptoms are best alleviated by *P. methysticum* treatment. The study involved 156 patients, and it utilized a five-item rating scale to assess twelve common symptoms associated with nervous anxiety, tension, and restlessness. Additionally, details were collected regarding the therapeutic setting, the perceived onset time of the effects, and the safety of the treatment. The treatment period for patients had a median duration of 28 days. Notably, all individual symptoms displayed significant and clinically meaningful improvements. The most remarkable results were observed in cases of nervous tension and restlessness, particularly in patients with acute symptoms rather than chronic ones. The study also determined that the application of kava was exceptionally safe, a conclusion drawn from the evaluation of laboratory data. To review, the study supports the efficacy and safety of short-term *P. methysticum* use in addressing “nervous anxiety, tension, and restlessness”, particularly in instances that are not chronic. These findings suggest that *P. methysticum* could be clinically beneficial for conditions like context-related phobias as per ICD-10 F40, nervous tension (ICD-10 R45.0), or restlessness and excitation (ICD-10 R45.1). However, a study demonstrated that this assumption is unfounded and should be corrected. The clinical evidence suggested that *P. methysticum* should be indicated for short-term situational anxiety, as defined by the German Commission E’s indication of “nervous anxiety, nervous tension and nervous restlessness”. This evidence also supported the safety of noble *P. methysticum* extracts, particularly regarding liver adverse events, as described by Kuchta et al. and Yadav et al., respectively [24,55]. 

In 2022, Kenda et al. reviewed that various *P. methysticum* extracts and isolated compounds have demonstrated interactions with GABAA receptors, inhibition of monoamine uptake through MAO-B inhibition and modulation of serotonin 5-HT1A receptors. In animal experiments involving rats and mice, diverse *P. methysticum* extracts and compounds displayed sedative, calming, and muscle-relaxing effects. Additionally, some studies indicated potential anticonvulsive, spasmolytic, neuroprotective, and analgesic properties. Clinical trials investigating the efficacy of *P. methysticum* preparations for anxiety disorders have yielded mixed outcomes. One randomized, double-blind controlled trial involving 135 participants in the kava-kava group and 135 participants in the placebo group revealed enhancements in anxiety symptoms and sleep. However, no significant distinctions between the groups were noted, implying that *P. methysticum* did not provide more significant relief than the placebo. Another analysis encompassing a combined sample from three randomized, double-blind controlled trials did not reveal any improvement in the kava-kava treatment group. Notably, there was an absence of observed hepatotoxicity in individuals treated with *P. methysticum*. In contrast, a separate randomized, double-blind, controlled study demonstrated a noteworthy reduction in anxiety within the *P. methysticum* treatment group. This effect was particularly pronounced among individuals experiencing moderate to severe generalized anxiety disorder. Genetic polymorphisms in GABA transporters rs2601126 and rs2697153 were linked to this outcome. However, the *P. methysticum* treatment group exhibited an increased incidence of headaches, while no discernible differences in liver function tests emerged between treatment groups. Overall, the clinical trials assessing *P. methysticum*’s effectiveness in generalized anxiety or anxiety during (peri)menopause, as reviewed by the European Medicines Agency in 2016, exhibited significant limitations. These included the trials’ brevity and inadequate follow-up periods, heterogeneity in the anxiety-afflicted population, insufficient data on the proportion of responders, variations in supplement extraction methods, diverse reference compounds, and discrepancies in dosages employed [56].

#### 2.2.7. *Pipturus albidus* A.Gray ex H.Mann (Hawaiian Name: Māmaki)

*Pipturus albidus* (māmaki) is an endemic Hawaiian plant of the Urticaceae family or Nettle family. Its medicinal properties are not well known, and it only grows on the Hawaiian Islands. It possesses antibacterial and antiviral properties and has been traditionally used for various medicinal purposes, including antioxidant effects, mild natural laxative properties, anti-allergic effects, promoting cardiovascular and liver health, and reducing stress levels [57]. Furthermore, the leaves of *P. albidus* are utilized in the preparation of herbal tea, which is believed to possess healing properties. These leaves are typically steeped in water, either fresh or dried, but boiling should be avoided as it could reduce the effectiveness of its effects. Traditionally, *P. albidus* tea has been used to treat a variety of issues, such as stress, anxiety, allergies, and regulation of blood pressure and cholesterol. In addition to its medicinal uses, the bark of *P. albidus* serves as an alternative to wauke (paper mulberry) when making kapa or bark cloth, and its roots are used for natural dye. Lastly, *P. albidus* fruit is located beneath the leaves of the plant [58].

#### 2.2.8. *Rhodiola rosea* L. (Introduced to Hawaii; Traditional Chinese Medicine (TCM): Hóng Jǐng Tiān)

*Rhodiola rosea*, commonly referred to as “roseroot”, “golden root” or “arctic root”, is a medicinal plant belonging to the Crassulaceae family. It has been used in traditional and popular medicine in many European and Asian countries [59,60]. In 2019, Yu et al. investigated the effects of *R. rosea* on oxidative stress, anxiety, and depression in patients with obstructive sleep apnea (OSA). Ninety patients diagnosed with moderate to severe obstructive sleep apnea (OSA) and exhibiting negative emotions based on polysomnography (PSG) as well as self-assessment scales for depression (SDS) and anxiety (SAS) were enrolled from the respiratory department of our hospital between February 2015 and February 2018. Employing a random number table method, the patients were allocated into three groups: the non-invasive ventilator group, the *R. rosea* + non-invasive ventilator group, and the *R. rosea* group, with each group containing 30 patients. The non-invasive ventilator group received continuous positive airway pressure (CPAP) treatment for 3 months, *R. rosea* + non-invasive ventilator group received oral *R. rosea* capsules in addition to CPAP, and *R. rosea* treatment group received only oral *R. rosea* capsules for 3 months. Changes in SDS and SAS scores were measured before and after the interventions, and alterations in serum superoxide dismutase (SOD) and malondialdehyde (MDA) levels were assessed using enzyme-linked immunosorbent assays. Results showed no significant differences in SDS and SAS scores among the three groups (*p* > 0.05). However, *R. rosea* + non-invasive ventilator group exhibited decreased SDS and SAS scores after treatment (*p* < 0.05) compared to the non-invasive ventilator group. Conversely, the *R. rosea* treatment group experienced increased SDS and SAS scores after treatment (*p* < 0.05). Notably, when comparing the *R. rosea* + non-invasive ventilator group to the *R. rosea* treatment group after treatment, the former demonstrated reduced SDS and SAS scores (*p* < 0.05). Initially, there were no significant differences in serum SOD and MDA levels among the three groups (*p* > 0.05). However, post-treatment, all groups exhibited elevated SOD levels and decreased MDA levels (*p* < 0.05). Furthermore, in comparison to the non-invasive ventilator group after treatment, the *R. rosea* + non-invasive ventilator group displayed decreased MDA levels in patients with elevated serum SOD levels (*p* < 0.05), as did the *R. rosea* treatment group (*p* < 0.05). Ultimately, the *R. rosea* + non-invasive ventilator group, when compared with *R. rosea* treatment group, showed decreased levels of serum SOD and MDA after treatment (*p* < 0.05). The study found that *R. rosea* may improve negative emotions such as anxiety and depression by inhibiting oxygen free radicals and lipid peroxidation in patients with OSA [61]. 

In 2019, Dinel et al. reported that examined the impact of repeated doses of a hydroethanolic root extract (HRE) from *R. rosea* on the hypothalamic-pituitary-adrenal (HPA) response in a murine model subjected to mild acute stress, as well as elucidate the underlying mechanisms. The experiment involved Balb/c mice administered either HRE (5 g/kg) or a control substance orally for two weeks before undergoing a mild acute stress regimen (consisting of open-field and elevated plus maze tests). Levels of corticosterone, a stress hormone, were assessed in plasma samples obtained from the mandibular vein prior to stress initiation, as well as 30, 60, and 90 min thereafter. At the 90 min mark, mice were euthanized, and the hippocampus, prefrontal cortex, and amygdala were harvested for high-frequency RT-PCR analysis to evaluate gene expression patterns. Results demonstrated that, following the introduction of mild acute stress, mice treated with HRE exhibited lower corticosterone levels after 30 min compared to the control group. Notably, the corticosterone levels in the HRE-treated stressed mice resembled those in non-stressed mice from the HRE group. Additionally, administration of HRE led to specific alterations in gene expression in various brain regions, particularly the hippocampus and prefrontal cortex. These genetic changes encompass stress-responsive genes related to neuronal architecture, activation of the HPA axis, and circadian rhythm. Collectively, the findings underscore the potential utility of *R. rosea* HRE in regulating reactivity to mild acute stress, as evidenced by its ability to diminish corticosterone levels and enhance the expression of stress-responsive genes, especially within the hippocampus and prefrontal cortex. This upregulation by *R. rosea* hydroethanolic root extract was associated with damping of corticosterone secretion and a faster return to the basal profile, indicating improved adaptation of the animals receiving *R. rosea* hydroethanolic root extract to a new environment. Further research is needed to understand the signaling pathways and transcription factors involved, as well as the impact of hydroethanolic root extract under stress conditions [62]. 

*R. rosea* supplementation may have beneficial effects on mild to moderate depression, mild anxiety, and mood in clinical trials. The therapeutic effects of *R. rosea* were observed to align with the specific medical conditions of the participants. Individuals with mild anxiety experienced a reduction in anxiety symptoms along with an uplift in mood. Those dealing with mild to moderate depression exhibited a decrease in depressive symptoms. In the case of individuals with major depression, there was a reduction in depression symptoms, though not to the same extent as seen with participants treated with sertraline, a conventional antidepressant. This suggests that *R. rosea* might be less effective in treating major depression compared to standard antidepressants. Additionally, individuals experiencing fatigue and treated with *R. rosea* showed improvements in fatigue symptoms but not in depression. Similarly, participants undergoing mentally stressful situations reported a decrease in mental fatigue and an increase in overall well-being, as summarized by Konstantinos et al. in 2020 [63]. Further research with randomized controlled trials is needed to confirm these effects. 

The use of *R. rosea* as a medicinal treatment for stress-induced conditions and disorders has been validated by extensive research, making it an effective remedy for stress-related issues. *R. rosea* herbal preparations have demonstrated promising results in treating mild-to-moderate depression and generalized anxiety. Traditional uses of *R. rosea* for stress and mood-related issues align with its potential to modulate anxiety and mood by hindering physiological stress responses. Clinical trials have indicated the effectiveness of *R. rosea* in reducing symptoms of generalized anxiety disorder and mild anxiety. Additionally, studies have shown that *R. rosea* can effectively alleviate symptoms of mild to moderate depression. Its antidepressant effects have been compared to conventional antidepressants like sertraline, with *R. rosea* showing similar odds of improvement and better tolerability. Combining *R. rosea* with sertraline has also led to improved outcomes in patients with major depressive disorder. Preclinical research suggests that *R. rosea*’s mechanisms involve influencing neuropeptide-Y expression, gene regulation related to behavior and mood, modulation of stress response pathways, and inhibition of monoamine oxidases. Moreover, *R. rosea* has shown potential for enhancing serotonin levels and neural stem cell proliferation in the hippocampus. Overall, these findings support the notion that *R. rosea* holds promise as a natural remedy for anxiety and depression, offering potential benefits and a more favorable risk-to-benefit ratio compared to certain conventional antidepressants, as described by Ivanova et al. in 2022 [60].

#### 2.2.9. *Stachytarpheta cayennensis* (Rich.) Vahl (Introduced to Hawaii; Hawaiian Name: Ōi or Ōwī)

The anti-anxiety and sedative effects of the methanolic leaf extract of *S. cayennensis* (Verbenaceae), its *n*-butanol, and aqueous fractions were demonstrated in mice, using rearing and spontaneous locomotion inhibition, pentobarbitone-induced sleep time prolongation, and elevated plus maze tests. The anxiolytic properties were evident in both the aqueous and *n*-butanol fractions at doses of 20 and 5 mg/kg, as indicated by the elevated plus maze test, as reported by Shah in 2021 [64].

**Table 2:** Hawaiian plants with beneficial effects on anxiety.

Scientific Name	Part of Used	Effective Extract/Chemical	Properties	References
*Azadirachta indica*	Leaves	Methanol extracts	Antidepressant activity	[31]
	Flowers	Aqueous extracts	Anxiolytic and antidepressant in rats	[32]
*Cocos nucifera*	Endocarp	Ethanol extracts	Anti-anxiety effects	[33]
	NA *	Oil extracts	Decreased levels of interleukin-6 (IL-6), state anxiety, and functional disability in patients	[34]
*Curcuma longa*	NA *	Curcumin	Neuroprotective effects against stress-induced toxicity and antidepressant activity by inhibiting the expression of Monoamine oxidase enzymes	[35]
	NA *	Curcumin	Improved brain-derived neurotrophic factor (BDNF) level in a mouse model of chronic stress	[36]
	NA *	Curcumin	Effective in treating depression	[37]
	NA *	Curcumin	Anxiolytic and antidepressant-like effects	[38]
	NA *	Extracts	Decreased anxiety levels in young, overweight and obese women	[39]
	NA *	Curcumin	Improve depressive and anxiety behavior	[40]
	NA *	Extracts	Improvements in digestive complaints and anxiety levels	[41]
	NA *	Curcumin	Decreased anxiety in rodent models	[42]
	NA *	Curcumin/ Extracts	Treatment for depression in animal and human trials	[43]
*Morinda citrifolia*	Fruits	Juices	Antipsychotic activity	[46]
	Fruits	Extracts	Anxiolytic, nootropic, and anti-craving activities	[47]
*Passiflora edulis*	Leaves	Hydroalcoholic and aqueous extracts	Anxiolytic and mild sedative effects in mice and rats	[50,51]
	Aerial parts	Butanol, methanol, ethanol, hydroethanolic, aqueous extracts	Anxiolytic-like effects in rat models	[48]
	NA *	Extracts	Anxiolytic-like effects in rat models	[52]
*Piper methysticum*	Roots	Aqueous extracts	Perform a larger, longer-term trial assessing the efficacy and safety in the treatment of generalized anxiety disorder	[53]
	NA *	Extracts	Therapeutic for anxiety and psychotic symptoms	[54]
	NA *	Extracts	Short-term situational anxiety	[24,55]
	NA *	Extracts	Reduction in anxiety in clinical trials	[56]
*Pipturus albidus*	Leaves	Aqueous extracts	Anxiety and reducing stress levels	[57,58]
*Rhodiola rosea*	NA *	Extracts	Improve anxiety and depression	[61]
	Roots	Hydroethanolic extracts	Decreased corticosterone levels and increased the expression of stress-responsive genes	[62]
	NA *	Extracts	Beneficial effects on mild to moderate depression, mild anxiety, and mood	[63]
	NA *	Extracts	Effective remedy for stress-related issues in pre-clinical trials and clinical studies	[60]
*Stachytarpheta cayennensis*	Leaves	Methanolic extracts	Anti-anxiety and sedative effects	[64]

* NA: Not available.

### 2.3. Hawaiian Plants with Beneficial Effects on Mood (Table 3)

#### 2.3.1. *Aloe vera* (L.) Burm.f. (Hawaiian Name: Panini ‘Awa‘awa)

*Aloe vera* (Asphodelaceae or Liliaceae) is a cactus-like plant that grows in hot, dry climates. It is cultivated in subtropical regions around the world, including Hawaii. The effects of *A. vera* on cells that produce dopamine were investigated by Tavakol in 2022. The findings revealed that an increase in focal adhesion kinase (FAK) activity coincided with a decrease in the Bax/Bcl2 ratio, the generation of reactive oxygen species (ROS), and minor changes in the sub-G1 phase of the cell cycle. These changes were accompanied by an elevation in glial cell-derived neurotrophic factor (GDNF), but no significant alteration was observed in brain-derived neurotrophic factor (BDNF), which is associated with depression and the recovery of motor impairments. These combined effects seemed to enhance the survival of dopaminergic cells, promote the extension of neurites, and reduce the number of unhealthy cells. However, it was noted that there was an increase in the expression of neuronal nitric oxide synthase (nNOS), leading to the production of nitric oxide (NO) and lactate dehydrogenase (LDH). Importantly, computational analyses of the interaction between the active components of *A. vera* and the catalytic domain of tyrosine hydroxylase (TH) demonstrated a robust binding affinity, even surpassing that of the original substrate, *L*-tyrosine. Subsequent to these computational findings, *A. vera* was found to suppress TH protein expression, consequently reducing dopamine levels. In summary, it can be proposed that *A. vera* has a positive impact on symptoms related to Parkinson’s disease by boosting factors that prevent cell death and encourage nerve growth. However, it also appears to inhibit TH and dopamine production, which could be likened to a Trojan horse strategy, ultimately leading to the worsening of disease symptoms over time. These outcomes suggest that the active constituents of Aloe vera could potentially be explored by pharmaceutical companies as promising agents for addressing neurological and psychiatric conditions, particularly in patients with elevated dopamine levels. *A. vera* has been hypothesized to enhance antiapoptotic markers and neurotrophic factors while suppressing dopamine levels. The results of a study provided an opportunity for pharmaceutical companies to use active components of *A. vera* as putative agents in neurological and psychiatric disorders and diseases to lower dopamine levels in patients with elevated dopamine levels, as described [65].

#### 2.3.2. *Morinda citrifolia* L. (Noni, Indian Mulberry, Great Morinda, Cheese Fruit)

*Morinda citrifolia* (Rubiaceae), commonly known as noni, is a perennial plant originating in Southeast Asia, and is now widely distributed in the Hawaii Islands. Scopoletin, a principal coumarin found in *M. citrifolia* fruits, has been known to possess analgesic properties and has the potential to enhance the function of the pineal gland, leading to increased production of serotonin within the brain, as reported by Almeida et al. in 2019 and Asiseh et al. in 2020, respectively [44,66]. In 2021, Ramya et al. investigated the impact of a methanolic extract from *M. citrifolia*, along with its bioactive constituents rutin and scopoletin (Figure 7). Within the MMC extract, the quantities of scopoletin and rutin were measured at 18.95 μg/mg and 1.66 μg/mg, respectively. Traditional antidepressants like tricyclic antidepressants (TCAs), selective serotonin reuptake inhibitors (SSRIs), selective noradrenaline reuptake inhibitors (SNRIs), and monoamine oxidase inhibitors (MAOIs) are effective for treating depression. Polynesians have historically employed *M. citrifolia* fruit in addressing depressive conditions. This study aims to enhance the existing understanding of the antidepressant-like effects of *M. citrifolia* fruit. It employs various preclinical mouse models of anxiety and depression, including the elevated plus maze (EPM), light/dark test (LDT), and tail suspension test (TST). Significantly, this research also takes preliminary steps to uncover the potential mechanism of action of *M. citrifolia* fruit by utilizing these animal behavioral models to explore its antidepressant-like behaviors. The results suggested that *M. citrifolia* fruit may be effective in treating depression, providing an important step forward in understanding the potential of this natural remedy [46].

Research has long supported the association between negative mood and increased alcohol use. In 2020, Khan and Pandy demonstrated the inhibitory activity of metformin (MMC) and acamprosate (ACAM) on ethanol-seeking behavior in a modified straight alley runway model. During the conditioning phase, animals were exposed to increasing doses of ethanol (ranging from 0.5 to 4 g/kg, administered intraperitoneally) for a span of 5 days (from Day 1 to Day 5). Subsequently, the extent of ethanol-seeking behavior was evaluated on the sixth day (termed the post-conditioning test). The results indicated that the group of mice exposed to ethanol showed clear signs of ethanol-seeking behavior, as evidenced by a marked decrease in the time taken to reach the intended target area. Notably, the administration of methanolic extract of *M. citrifolia* in various doses (1 g/kg, 3 g/kg, and 5 g/kg, given orally) led to a significant reversal of ethanol-seeking behavior, analogous to the effects observed with the reference drug acamprosate (ACAM; administered at 300 mg/kg, orally) in mice. Following the post-conditioning test, a series of non-rewarded extinguishing trials were conducted over a period of five days (ranging from Day 7 to Day 11). After five days of abstaining from ethanol, the reintroduction of a priming dose of ethanol (equivalent to one-fifth of the highest dose applied during the control phase, i.e., 0.8 g/kg, administered intraperitoneally) within the mice’s home cages significantly reinstated the inclination for ethanol-seeking behavior. Of particular interest, when administered at a higher dose (5 g/kg, orally), methanolic extract of *M. citrifolia* demonstrated a noteworthy ability to prevent ethanol-induced reinstatement in mice, akin to the effects observed with the reference drug ACAM (300 mg/kg, orally). These findings underscore the potential of the methanolic extract of *M. citrifolia* to mitigate the seeking behavior associated with ethanol in a modified mouse runway setup, suggesting its potential as an effective therapeutic candidate for addressing alcohol dependence. The findings suggest that MMC and ACAM have a dopaminergic activity that can reduce the rewarding effects of ethanol, thus reducing ethanol-seeking behavior. Previous studies have shown that the ethyl acetate fraction of the methanolic extract of unripe *M. citrifolia* fruit has a biphasic effect on the dopaminergic system in mice. When administered at lower doses (<25 mg/kg, orally), it exhibited the ability to reduce cage climbing induced by the dopamine receptor agonist apomorphine and stereotypic behavior induced by methamphetamine in mice. Conversely, at a higher dose (3000 mg/kg, orally), ethyl acetate extract of *M. citrifolia* inhibited catalepsy induced by haloperidol (0.5 mg/kg, intraperitoneally), a dopamine D2 receptor antagonist, in mice. Conversely, at a higher dose, it inhibited the effects of a dopamine receptor antagonist. The study suggested that ethyl acetate extract of *M. citrifolia*’s biphasic effects might be due to its interaction with different types of dopaminergic receptors, blocking them in distinct ways. This research provides insights into how noni fruit extract affects the dopaminergic system in a dosage-dependent manner. Furthermore, *M. citrifolia* fruit has been proposed to possess antidopaminergic properties, potentially leading to anti-motivational effects against ethanol self-administration in mice. The phytochemicals rutin and scopoletin (Figure 7) may be responsible for this effect and are currently being studied to further explore their potential against ethanol-seeking behavior in mouse models [67]. 

Patients with obsessive-compulsive disorder (OCD) often experience aversive emotions. In 2022, Jeyabalan et al. investigated the antidepressant and sedative-like effects of *M. citrifolia* fruit extract (MCFE). An investigation was carried out on brain neurotransmitters such as dopamine (DA), serotonin, and noradrenaline (NA). Each of the distinct groups accommodated five mice, and the treatment was administered to the animals for a span of 15 days. The marble burying test was scrutinized for a duration of 30 min on days 1, 7, and 14, while the nestlet shredding test was evaluated over a 60-min period on days 2, 8, and 15. The application of MCFE (at doses of 100 and 200 mg/kg, administered orally) exhibited notable enhancements in both behavioral tasks compared to the control group. Additionally, the administration of diazepam (at a dose of 2 mg/kg, intraperitoneally) and fluoxetine (at a dose of 15 mg/kg, administered orally) also resulted in significant improvements in both tasks when compared to the control mice. Subsequent investigation into locomotor activity revealed that MCFE and fluoxetine did not induce any alterations in locomotor functions when contrasted with mice treated with the vehicle. Conversely, diazepam displayed a substantial reduction in locomotion compared to the control group. Significant enhancements in biogenic amines were observed in the animals treated with MCFE, with a noticeable elevation in serotonin levels. Examination of brain, liver, and kidney tissues following MCFE administration exhibited intact morphological structures devoid of any signs of toxicity or irregularities. Taken together, these findings strongly suggest that MCFE holds potential as a viable drug candidate for addressing OCD. Future research endeavors should prioritize the identification of constituents within *M. citrifolia* and their anti-compulsive properties. The results showed that MCFE had anti-OCD-like activity in a dose-dependent manner and inhibited marble burying and nestlet shredding behavior without affecting locomotion. The findings suggested that MCFE produced its anti-OCD effects by interacting with the serotonergic, noradrenergic, and dopaminergic systems, as evidenced by its ability to restore dopamine and serotonin levels and reduce inflammation in the brain. Further research is needed to evaluate the safety and efficacy of MCFE in preclinical and clinical studies [68].

**Table 3:** Hawaiian plants with beneficial effects on mood.

Scientific Name	Part of Used	Effective Extract/Chemical	Properties	References
*Aloe vera*	NA *	NA *	Enhance antiapoptotic markers and neurotrophic factors	[65]
*Morinda citrifolia*	Fruits	Scopoletin	Regulate serotonin levels in the body	[44,66]
	Fruits	Extracts	Antidepressant effects in animal models	[46]
	Fruits	Methanolic extract	Anti-motivational effects against ethanol self-administration in mice	[67]
	Fruits	Extracts	Antidepressant and sedative-like effects	[68]

* NA: Not available.

### 2.4. Hawaiian Plants with Beneficial Effects on Stress/Pain (Table 4)

#### 2.4.1. *Argyreia speciosa* (L.f.) Sweet

In 2022, Verma et al. conducted a review of research regarding the potential anti-stress properties of a hydroalcoholic root extract of *A. speciosa*. The review included studies that used swimming endurance tests, anoxic tolerance tests, and cold restraint stress tests in mice and rats. Results indicated that *A. speciosa* had a significant anti-stress effect [69].

#### 2.4.2. *Curcuma longa* L. (Tumeric, Indian Saffron, Hawaiian Name: Ōlena)

Curcumin has been found to attenuate cancer-induced bone pain by up-regulating the expression of proopiomelanocortin in primary rat dorsal root ganglion neurons and promoting the release of β-endorphin and enkephalin. Investigation of the pain-relieving effectiveness and mechanism of curcumin in relieving pain caused by cancer-induced bone issues. These findings demonstrated that consistent administration of curcumin at varying doses (30, 60, 120 mg/kg, injected twice daily for 11 days) led to significant pain relief, although it did not influence the progression of bone cancer pain itself. Importantly, when the opioid receptor antagonist naloxone was administered prior to curcumin treatment, it substantially reversed the pain-relieving effects induced by curcumin. Additionally, in laboratory-cultured primary rat dorsal root ganglion (DRG) neurons, curcumin notably increased the expression of proopiomelanocortin (Pomc) and facilitated the release of β-endorphin and enkephalin. Furthermore, pretreatment with antibodies targeting β-endorphin or enkephalin significantly reduced the pain relief induced by curcumin in cases of cancer-induced bone pain. This study is the first to demonstrate curcumin’s ability to alleviate pain associated with cancer-induced bone issues. The results also indicate that the enhanced expression of DRG neuron β-endorphin and enkephalin contributes to curcumin’s pain-relieving effects in conditions characterized by heightened pain sensitivity. This is the first time that curcumin has been shown to have an antinociceptive effect in pain hypersensitivity conditions, and the effects may be mediated by increased expression of β-endorphin and enkephalin which was demonstrated by Zhao et al. in 2021 [70].

#### 2.4.3. *Morinda citrifolia* L. (Noni—Indian Mulberry, Great Morinda, Cheese Fruit)

*Morinda citrifolia* has been found to have numerous beneficial properties, including its ability to enhance the mucosal defensive mechanisms of the body. Studies have demonstrated that *M. citrifolia* worked by suppressing serotonin, free radicals, and cytokine-mediated inflammation. It also exhibits a dopamine antagonist effect and hence was used as an antipsychotic, all of which were known to contribute to inflammatory responses and potential health risks. Furthermore, *M. citrifolia* exhibits the capacity to hinder the creation of pro-inflammatory cytokines and showcases calming attributes akin to narcotic substances. These qualities collectively contribute to its analgesic impact in cases of dysmenorrhea, as reported by Lohani et al. [71]. A study investigating the effect of *M. citrifolia* extract and Tahitian noni juice on locomotion in mice showed no significant difference compared to the control group. Administration of *M. citrifolia* extract at doses of 0.5, 1, 3, and 5 g/kg and Tahitian noni juice 100% available as a source of drinking water for 24 h prior to testing had no effect on locomotion or behavior. No toxic effects were observed, and no mortality or changes in behavior occurred. Furthermore, the extract derived from the roots of the *M. citrifolia* fruit has shown sedative and analgesic effects in mice. Researchers have suggested that these effects may be linked to the activation of central opioid receptors. These findings suggested that *M. citrifolia* extract and Tahitian noni juice are safe and can be used for further neuropharmacological research, as mentioned by Khan et al. in 2022 [72].

**Table 4:** Hawaiian plants with beneficial effects on stress/pain.

Scientific Name	Part of Used	Effective Extract/Chemical	Properties	References
*Argyreia speciosa*	Roots	Hydroalcoholic extracts	Antistress effect in mice	[69]
*Curcuma longa*	NA *	Curcumin	Antinociceptive effect in pain hypersensitivity conditions	[70]
*Morinda citrifolia*	Fruits/flowers	NA *	Exhibits dopamine antagonist effect	[71]
	Fruits/ roots	Extracts	Locomotion effect in mice Sedative and analgesic effects	[72]

* NA: Not available.

The most relevant finding in this review is that Hawaiian plants have the potential to provide beneficial effects for a wide range of conditions, including sleep, mood disturbances, anxiety, stress, and pain. The review highlights the historical medicinal use of endemic plants in Hawaii and their importance in traditional Hawaiian healing practices. It suggests that reintegrating the use of these plants into modern healthcare could offer potential benefits for improving health and well-being in current generations. This manuscript underscores the significance of indigenous plants and their potential contributions to addressing various health concerns.

## 3. Conclusions

This review summarized the studied effects of Hawaiian plants on sleep, anxiety, mood, stress, and pain. The evidence suggests that there are a number of Hawaiian plants that have potentially beneficial effects on these biological processes. Six Hawaiian plants (*A. koa*, *C. longa*, *D. viscosa*, *M. pudica*, *P. methysticum*, and *S. cayennensis*) have been shown to have potentially beneficial effects on sleep (Table 1). These plants contain compounds that have sedative effects in animals. Nine Hawaiian plants (*A. indica*, *C. nucifera*, *C. longa*, *M. citrifolia*, *P. edulis*, *P. methysticum*, *P. albidus*, *R. rosea*, and *S. cayennensis*) have been shown to have potential anxiolytic effects (Table 2). *A. indica* leaf extracts, *C. nucifera* endocarp extracts, *M. citrifolia* juice, *P. edulis* leaf extracts, *P. albidus* leaf tea, *R. rosea* extracts, and *S. cayennensis* leaf extracts, curcumin, and kavalactones have all been shown to have anti-anxiety effects in animal models. In addition, curcumin has been shown to have antidepressant effects in both animal and human studies. Since depression and anxiety often coexist, *C. longa* and curcumin could help reduce both symptoms. Two Hawaiian plants (*A. vera* and *M. citrifolia*) have been shown to have potentially beneficial effects on mood (Table 3). *A. vera* can improve overall mood and has been shown to enhance anti-apoptotic markers and neurotrophic factors while suppressing dopamine levels. *M. citrifolia* has been studied for its potential antidepressant effects and has been shown to have antidopaminergic properties and anti-obsessive-compulsive disorder (OCD)-like activity. Three Hawaiian plants (*A. speciosa*, *C. longa*, and *M. citrifolia*) have been shown to have potential therapeutic benefits for stress and pain (Table 4). *A. speciosa* has been shown to have an anti-stress effect in mice and rats, *C. longa* has been shown to attenuate cancer-induced bone pain, and *M. citrifolia* has been shown to have numerous beneficial properties, including its ability to enhance the mucosal defensive mechanisms of the body and to suppress serotonin, free radicals, and cytokine-mediated inflammation. The three Hawaiian plants with the most potential for therapeutic benefits are *A. speciosa*, *C. longa*, and *M. citrifolia*. These plants have been shown to have anti-stress, anti-anxiety, and antidepressant effects in animal models. More research is needed to fully understand their safety and efficacy, but these plants may be promising candidates for the development of new therapeutic interventions.

## 4. Perspectives

This review emphasizes *A. speciosa*, *C. longa*, and *M. citrifolia* as notable candidates for therapeutic applications. The identification of these botanical species suggests avenues for innovative interventions that may provide natural alternatives or supplementary approaches for individuals dealing with mental health disorders, stressors, and pain. The varied effects exhibited by these Hawaiian plants align with holistic medical paradigms, which consider the intricate interplay of physiological and psychological dimensions. This prompts further exploration of comprehensive strategies capable of addressing the multifaceted aspects of human health. Leveraging the potential health benefits of Hawaiian plants necessitates a comprehensive understanding of their traditional uses, chemical constituents, and prior research outcomes. To establish their safety and efficacy as treatments, robust clinical trials are imperative, given their status as the gold standard in research. In addressing conditions such as insomnia, anxiety, mood disorders, and stress-induced pain, future investigations should prioritize the rigorous implementation of clinical trials. Furthermore, identifying the bioactive compounds responsible for the therapeutic effects of Hawaiian plants is pivotal for creating standardized extracts suitable for use in clinical studies. Exploring the underlying mechanisms of action of these botanicals is also a critical avenue for future research, aiding in the development of more potent and convenient therapeutic formulations. The formulation can encompass various modalities of delivering the therapeutic compounds found in Hawaiian plants to individuals for treatment purposes. These formulations may include the development of standardized herbal extracts from Hawaiian plants containing the active compounds responsible for their therapeutic effects. These extracts can be administered in diverse forms, such as tinctures, capsules, or tablets. Topical preparations, such as creams or oils containing active compounds, can be developed for targeted application to the skin, particularly for pain relief or skin-related conditions. Preparing teas or infusions from the plants offers a traditional and straightforward method of delivering therapeutic compounds. Inhalable products, such as essential oil diffusions, can be developed for delivering aromatic compounds that may have mood-enhancing and stress-relieving effects. Dietary supplements with concentrated amounts of active compounds can be designed to support mental well-being. Formulations that combine extracts from multiple Hawaiian plants could potentially enhance their synergistic effects. It is important to note that while these formulations can encompass various natural products, the passage does not explicitly address whether the focus is on developing pharmaceutical formulations adhering to stringent regulatory standards or on more traditional and holistic treatment approaches. Ensuring the enduring safety of Hawaiian plants requires extended clinical trials involving repeated administration of herbal remedies. Continuous monitoring of participants over extended periods will help detect potential adverse effects or interactions with other medications.

Collaboration among traditional practitioners, scientists, and healthcare professionals is paramount to bridging the gap between traditional knowledge and contemporary research. Such collaboration may facilitate the development of culturally suitable and effective treatments derived from Hawaiian plants, benefiting local communities and the broader population grappling with these common health challenges.

In conclusion, the review underscores the intriguing potential of Hawaiian plants in providing intrinsic solutions for sleep-related issues, anxiety, mood disorders, stress, and pain. Despite promising initial findings, additional extensive research is essential to firmly establish their safety, efficacy, and mechanisms of action. These botanical species potentially open a novel pathway for developing therapeutic interventions aligned with a holistic understanding of health and well-being.

## 5. Methodology

**Figure 8:**
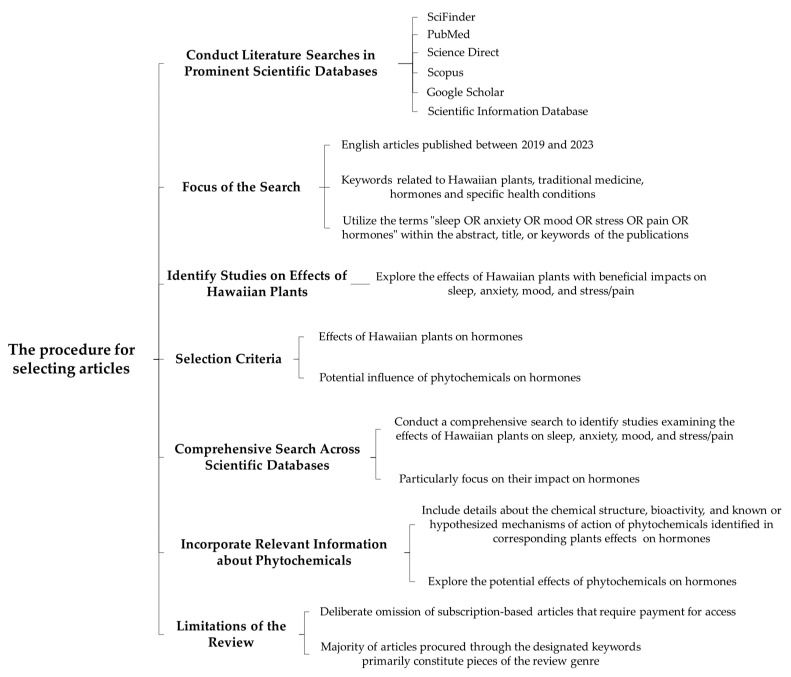
The procedure for selecting articles.

The article selection process (Figure 8) involved exhaustive searches across respected scholarly databases: SciFinder, PubMed, Science Direct, Scopus, Google Scholar, and the Scientific Information Database. The focus was on English articles published between 2019 and 2023, using keywords related to Hawaiian plants, traditional medicine, hormones, and specific health conditions. The terms “sleep OR anxiety OR mood OR stress OR pain OR hormones” were used in abstracts, titles, or keywords. The goal was to identify studies exploring the effects of Hawaiian plants on sleep, anxiety, mood, and stress/pain. Selected articles prioritized those investigating the interaction between these plants and hormones, while also considering the impact of phytochemicals. This search meticulously examined research investigating the effects of Hawaiian plants on sleep, anxiety, mood, and stress/pain, particularly with respect to hormonal mechanisms. Furthermore, chosen articles provided in-depth insights into the chemical properties of phytochemicals, their biological activities, and their known or hypothesized influence on hormonal processes. It is important to note that subscription-based articles requiring payment were intentionally excluded. The majority of the gathered articles are anticipated to fall within the review genre.

The article selection procedure was based on the following criteria:

### 5.1. Conducting Literature Searches in Prominent Scientific Databases

Thorough searches in well-regarded scientific databases such as SciFinder, PubMed, Science Direct, Scopus, Google Scholar, and the Scientific Information Database were conducted.

### 5.2. Focus of the Search

The search targeted English articles released between 2019 and 2023. Keywords revolved around Hawaiian plants, traditional medicine, hormones, and specific health conditions. The search will incorporate the terms “sleep OR anxiety OR mood OR stress OR pain OR hormones” within publication abstracts, titles, or keywords.

### 5.3. Identification of Studies on Effects of Hawaiian Plants

The impacts of Hawaiian plants that positively affect sleep, anxiety, mood, and stress/pain are examined.

### 5.4. Selection Criteria

Articles will be chosen based on the effects of Hawaiian plants on hormones and the potential impact of phytochemicals on hormones.

### 5.5. Comprehensive Search across Scientific Databases

A thorough search was undertaken to locate studies investigating the effects of Hawaiian plants on sleep, anxiety, mood, and stress/pain. Particular attention was paid to their influence on hormones.

### 5.6. Inclusion of Relevant Phytochemical Information

Relevant details about the chemical makeup, bioactivity, and established or theoretical mechanisms of action of phytochemicals identified in the plants that impact hormones were incorporated. Additionally, the potential effects of these phytochemicals on hormones were explored.

### 5.7. Limitations of the Review

Articles that require payment for access will be intentionally excluded. The majority of gathered articles via the specified keywords will predominantly belong to the review genre.

The selection process involved applying these inclusion and exclusion criteria to the search results obtained from the mentioned databases. Articles meeting these criteria were then further evaluated for relevance, quality, and significance to determine the most appropriate ones to be included in the literature review.

## Data Availability

Data sharing is not applicable.
